# A high-resolution genomic analysis of multidrug-resistant hospital outbreaks of *Klebsiella pneumoniae*

**DOI:** 10.15252/emmm.201404767

**Published:** 2015-02-20

**Authors:** Hao Chung The, Abhilasha Karkey, Duy Pham Thanh, Christine J Boinett, Amy K Cain, Matthew Ellington, Kate S Baker, Sabina Dongol, Corinne Thompson, Simon R Harris, Thibaut Jombart, Tu Le Thi Phuong, Nhu Tran Do Hoang, Tuyen Ha Thanh, Shrijana Shretha, Suchita Joshi, Buddha Basnyat, Guy Thwaites, Nicholas R Thomson, Maia A Rabaa, Stephen Baker

**Affiliations:** 1 The Hospital for Tropical Diseases, Wellcome Trust Major Overseas Programme, Oxford University Clinical Research UnitHo Chi Minh City, Vietnam; 2 Patan Academy of Health Sciences, Wellcome Trust Major Overseas Programme, Oxford University Clinical Research UnitKathmandu, Nepal; 3 The Wellcome Trust Sanger InstituteHinxton, Cambridge, UK; 4 Addenbrooke's HospitalCambridge, UK; 5 Centre for Tropical Medicine, Nuffield Department of Clinical Medicine, Oxford UniversityOxford, UK; 6 MRC Centre for Outbreak Analysis and Modelling, Department of Infectious Disease Epidemiology, School of Public Health, Imperial CollegeLondon, UK; 7 The London School of Hygiene and Tropical MedicineLondon, UK; 8 Centre for Immunity, Infection and Evolution, University of EdinburghEdinburgh, UK

**Keywords:** antimicrobial resistance, bloodstream infections, carbapenemases, *Klebsiella pneumoniae*, nosocomial infections

## Abstract

Multidrug-resistant (MDR) *Klebsiella pneumoniae* has become a leading cause of nosocomial infections worldwide. Despite its prominence, little is known about the genetic diversity of *K. pneumoniae* in resource-poor hospital settings. Through whole-genome sequencing (WGS), we reconstructed an outbreak of MDR *K. pneumoniae* occurring on high-dependency wards in a hospital in Kathmandu during 2012 with a case-fatality rate of 75%. The WGS analysis permitted the identification of two MDR *K. pneumoniae* lineages causing distinct outbreaks within the complex endemic *K. pneumoniae*. Using phylogenetic reconstruction and lineage-specific PCR, our data predicted a scenario in which *K. pneumoniae*, circulating for 6 months before the outbreak, underwent a series of ward-specific clonal expansions after the acquisition of genes facilitating virulence and MDR. We suggest that the early detection of a specific NDM-1 containing lineage in 2011 would have alerted the high-dependency ward staff to intervene. We argue that some form of real-time genetic characterisation, alongside clade-specific PCR during an outbreak, should be factored into future healthcare infection control practices in both high- and low-income settings.

## Introduction

*Klebsiella pneumoniae* is one of the leading causes of hospital-acquired infections globally. These infections (typically pneumonia, urinary tract infections, bacteraemia and wound infections) are frequently severe, notoriously affecting incapacitated patients with a suppressed immune status and neonates in intensive care units (Podschun & Ullmann, 1998). *Klebsiella pneumoniae* are commonly resistant to multiple antimicrobials and are generally acknowledged to be a major source of antimicrobial resistance genes that can spread into other Gram-negative pathogens. This has led to multidrug-resistant (MDR) *K. pneumoniae* being recently singled out as an “urgent threat to human health” by the U.S. Centers for Disease Control and Prevention (CDC), the World Health Organization (WHO), the Government of the United Kingdom and other international healthcare organizations (Centres for Disease Control & Prevention, 2013; UK Department of Health & Department for Environment Food & Rural Affairs, 2013). *Klebsiella pneumoniae* has earned notoriety largely due to resistance to “last resort” antimicrobials such as 3^rd^ generation cephalosporins and carbapenems, which has significantly narrowed, or in some settings completely removed, the therapeutic options for the treatment of MDR *K. pneumoniae* infections (Munoz-Price *et al*, 2013).

The problem of antimicrobial resistance is highlighted by a recent increase of carbapenem-resistant *K. pneumoniae*, which has largely been driven by the emergence and spread of mobile genetic elements carrying carbapenemase resistance genes including the metallo-beta-lactamase NDM-1 (Nordmann *et al*, 2011; Tzouvelekis *et al*, 2012). Indeed, *bla*_NDM-1_ is now considered to be endemic in the Indian subcontinent and reports of NDM-1-producing *Klebsiella* from hospital- and community-acquired infections elsewhere indicate global dispersion (Walsh *et al*, 2011; Villa *et al*, 2012; Hudson *et al*, 2014). Perhaps of additional concern is the fact that *bla*_NDM-1_ is frequently coupled with several antimicrobial resistance determinants on the same conjugative plasmid, thus presenting a bleak therapeutic outlook for severe infections caused by *K. pneumoniae* carrying such plasmids.

The genetic diversity of *K. pneumoniae* causing hospital outbreaks has been studied previously, with the primary focus being particular emergent clones in affluent settings (Brisse *et al*, 2009; Snitkin *et al*, 2012; Deleo *et al*, 2014). There is currently limited knowledge regarding the overall genetic diversity of this major pathogen within a single hospital setting. This limitation is highly pertinent in resource-poor settings where these infections may exhibit more antimicrobial resistance and cause particularly aggressive infections. This knowledge gap is due in part to a lack of sensitive phenotypic and molecular tools for accurately differentiating or subtyping *K. pneumoniae*. For most Gram-negative bacteria, relatedness among isolates has traditionally been deduced using antimicrobial susceptibility patterns, crude microbiological methods or molecular typing methods such as pulsed field gel electrophoresis (PFGE) or multilocus sequence typing (MLST). Whilst providing some relevant information, these methods rarely provide sufficient discriminatory power for characterising and precisely tracking an outbreak. This is a major constraint that needs to be addressed if we are to make any substantial impact on understanding the genomics and epidemiology of such an important and neglected pathogen, especially if the current trajectory of antimicrobial resistance is sustained.

Here, we have used whole-genome sequencing (WGS) and state-of-the-art analytical techniques to define an alarming outbreak of MDR *K. pneumoniae* within a major hospital in Kathmandu, Nepal, during 2012. This outbreak occurred in children admitted to neighbouring high-dependency paediatric wards and was characterised by high incidence of bloodstream infections and a case-fatality rate of 75% (36/48). At the time, this was hypothesised to be an outbreak caused by a single strain, but we show that it was in fact two outbreaks triggered by two distantly related MDR *K. pneumoniae* lineages against a complex backdrop of sporadic endemic *K. pneumoniae* infections. This study provides essential insights into antimicrobial resistance, virulence, and pre- and post-outbreak strain persistence of an increasingly important global pathogen in a typical limited resource hospital in Kathmandu, Nepal.

## Results

### Clinical description of *Klebsiella pneumoniae* outbreak

**Figure 1:**
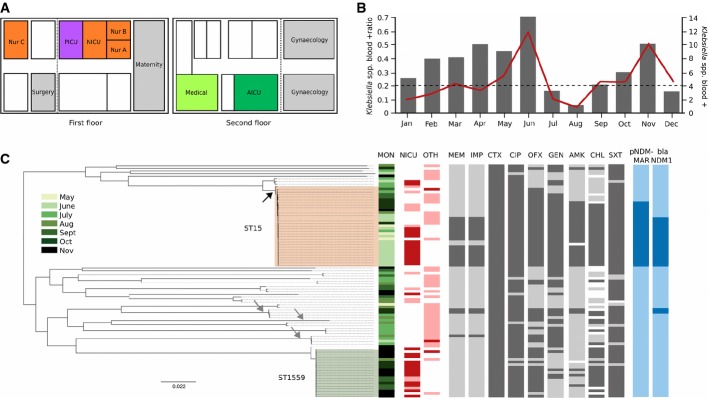
Hospital outbreaks of *Klebsiella pneumoniae* in Kathmandu, Nepal, in 2012
A Graphical representation of the layout of the wards at Patan Hospital affected by the 2012 *K. pneumoniae* outbreaks. The affected wards on the first and second floor (directly above) are highlighted in colour, which correspond with the sub-lineages indicated in Fig 2A; nurseries and neonatal intensive care unit (NICU), orange; paediatric intensive care unit (PICU), purple; adult intensive care unit (AICU), dark green; medical ward, bright green.B Plot (red line) shows the monthly ratio of *Klebsiella* spp. isolated from blood at Patan Hospital in 2012 with respect to *Klebsiella* spp. isolated from all other samples (primary *y*-axis). Broken line corresponds to the mean ratio of *Klebsiella* spp. isolated from blood in 2012. Histogram shows the number of *Klebsiella* spp. isolated from blood samples at Patan Hospital per month in 2012 (secondary *y*-axis).C Maximum likelihood phylogeny based on core genome SNPs of the 89 *K. pneumoniae* isolates with the corresponding metadata: month of isolation (MON) in 2012 (see key), ward (NICU, PICU and nurseries (NICU) or other (OTH)) and specimen type from which the organism was isolated (red, blood; pink, others), antimicrobial susceptibility profile (susceptible, light grey; non-susceptible, dark grey; and missing data, white) by disc diffusion against MEM (meropenem), IMP (imipenem), CTX (cefotaxime), CIP (ciprofloxacin), OFX (ofloxacin), GEN (gentamicin), AMK (amikacin), CHL (chloramphenicol) and SXT (trimethoprim/sulfamethoxazole). The presence (dark blue) or absence (light blue) of the pNDM-MAR-like plasmid and the *bla*_NDM__-1_ gene. The black arrow distinguishes the lineage with three mutations in genes associated with fluoroquinolone susceptibility (GyrA-S83F, GyrA-S87A, ParC-S80I). The grey arrows identify lineages with two mutations in genes associated with fluoroquinolone susceptibility (GyrA-S83I, ParC-S80I). The scale bar indicates the number of nucleotide changes per site (see Materials and Methods).

From May 2012, a proportional increase in the number of *Klebsiella* spp. bloodstream infections in children admitted to the several proximal high-dependency wards in Patan Hospital was reported (Fig1A). The specific wards affected were the nurseries, the neonatal intensive care unit (NICU) and the paediatric intensive care unit (PICU). Indeed, almost 60% (14/24) of the *Klebsiella* spp. isolated in the hospital in May/June 2012 were from blood, compared to an overall average of 20% (81/386) for 2012 (Fig1B). The bloodstream-associated *Klebsiella* isolates in May/June 2012 were confirmed as *K. pneumoniae* and were first noted to be cefotaxime resistant with variable susceptibility to carbapenems (9/14; 64%). An outbreak was suspected, and as a result, the NICU and the PICU were closed for maintenance (including the repair of leaking pipework) and fumigation (NICU closed, 4/7/12–24/7/12; PICU closed, 9/7/12–24/7/12). Later in 2012, we observed a secondary rise in the number of *K. pneumoniae* isolations in blood taken from children on the same high-dependency wards (50% (10/20) of all *Klebsiella* spp. isolated in the hospital in November 2012) (Fig1B). At this time, a recrudescence of the earlier outbreak was suspected, given that the *K. pneumoniae* originated from the same wards and were again resistant to cefotaxime.

On review, we found that 270 *Klebsiella* spp. isolates had been cultured across the hospital in 2012, of which 18% (48/270) were *K. pneumoniae* originating from blood cultures taken in the three nurseries, the NICU and the PICU (Fig1A). Of these 48 cases of childhood *K. pneumoniae* bloodstream infections, 36 (75%) died in hospital. To better understand the nature of this dual peaked *K. pneumoniae* outbreak, we randomly selected 90 hospital *K. pneumoniae* isolates between May and December 2012 for WGS, which included 29 suspected outbreak strains from blood (isolated from blood on the NICU, *n* = 8; PICU, *n* = 7; nurseries, *n* = 14) (isolate information shown in Supplementary Table S1).

### Genome sequencing of *Klebsiella pneumoniae* outbreak strains

The 90 selected *K. pneumoniae* strains were subjected to short-read Illumina sequencing. Read mapping to *K. pneumoniae* reference sequence MGH78578 showed an average of 91.12% genome coverage among 89 genomes, while one strain failed due to insufficient coverage. Reads were *de novo* assembled into contigs, and the pan and core genome were constructed by protein clustering (see Materials and Methods). The MLST gene alleles were extracted, and a phylogeny using the seven concatenated MLST gene sequences of all 89 strains in comparison to *K. variicola* (KpIII) and *K. pneumoniae* MGH78578 (KpI) showed that KpI was nested within the 89 hospital strains, while KpIII was clustered as an out-group to all others (unpublished observations). This indicated that all 89 strains isolated were related and also confirmed that they were all restricted to the *K. pneumoniae* species (Brisse *et al*, 2009).

We defined the core (common) genome of the 89 sequenced *K. pneumoniae* as the concatenation of 3,692 gap-free putative orthologous coding sequences, constituting 3,529,263 base pairs of DNA sequence. Since two of the genome sequences showed no evidence of plasmids (reference genome *K. pneumoniae* MGH78578), the core genome contained minimal sequences associated with mobile genetic elements. In this reference-free alignment, we identified 101,250 SNPs across all strains, with the pairwise disparity between strains ranging from 2 to 21,373 SNPs. Putative recombination loci were further detected and removed, and the final post-recombination removal alignment consisted of 83,859 variable SNP sites among the *K. pneumoniae*. This recombination-free SNP variation across the core genome was used to construct a maximum likelihood phylogenetic tree of all 89 sequenced *K. pneumoniae* (Fig1C) and revealed a structure dominated by two major genetically distinct lineages.

We next overlaid supplementary metadata onto the whole-genome phylogeny, which included the date of isolation, ward of admission, specimen type and antimicrobial susceptibility against meropenem, imipenem, cefotaxime, ciprofloxacin, ofloxacin, gentamicin, amikacin, chloramphenicol and trimethoprim/sulfamethoxazole (Fig1C). The first dominant lineage in this *K. pneumoniae* collection belonged to MLST-sequence type (ST)15 (*n* = 31), of which 17 were phenotypically resistant to carbapenems, which appeared to be associated with a pNDM-MAR plasmid containing the *bla*_NDM-1_ carbapenemase gene. Notably, these ST15 strains were resistant to all other antimicrobials tested (Fig1C). Fifteen of these ST15 strains were associated with bloodstream infections, and the majority were associated with the NICU complex (NICU, PICU and nurseries) (Fig1C). ST15 is an individual clonal group (CG) within the MLST clonal complex 14 (CC14) (Bialek-Davenet *et al* 2014), an internationally dispersed clonal complex that has previously been associated with hospital-acquired infections (Hrabák *et al*, 2009).

The second major lineage was found to be a novel sequence type (designated as ST1559), of which all 19 were carbapenem susceptible (variable susceptibility was observed against the other seven antimicrobials tested) (Fig1C). ST1559 is a single locus variant of ST152, with mutation in the *ton*B*-*56 allele (T261G). Fourteen of these ST1559 organisms were associated with bloodstream infections and were again mainly associated with the NICU complex (Fig1C). These data show that the increased incidence of *K. pneumoniae* bloodstream infections on the paediatric wards in Patan Hospital in 2012 was in fact due to two independent outbreaks caused by distinct and widespread lineages.

### Defining outbreak lineages

**Figure 2:**
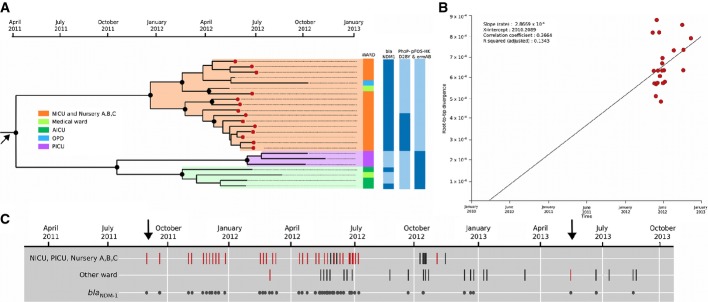
Temporal phylogenetic reconstruction and retrospective PCR detection of *Klebsiella pneumoniae* Outbreak Lineage 1
A Bayesian phylogenetic reconstruction of Outbreak Lineage 1 using the core genome SNPs of 24 isolates with a corresponding temporal timescale. The black filled circles indicate a posterior probability > 0.8 on internal nodes. The red filled circles indicate *K. pneumoniae* isolates cultured from blood. The ward from which each specimen was collected: orange (NICU and nurseries A, B, C), dark green (AICU), light green (medical ward), light blue (OPD, outpatient department) and purple (PICU). The presence (dark blue) and absence (light blue) of specific genetic determinants for each of the 24 isolates, including *bla*_NDM__-1_, are highlighted.B Linear regression plot of the date (*x*-axis) against the root-to-tip divergence (*y*-axis) used to indicate correspondence between phylogenetic and temporal signal. Estimation of temporal signal was performed in Path-O-Gen v1.4 for the core genome SNP-based maximum likelihood phylogeny of 24 Outbreak Lineage 1 *K. pneumoniae*. Additional estimated parameters are inset.C The temporal distribution of 70 PCR-confirmed Outbreak Lineage 1* Klebsiella pneumoniae* infections by their ward of isolation. First row: outbreak isolates originating from the NICU, PICU and the nurseries. Second row: isolates originating from other wards, including the AICU, gynaecology ward, maternity ward, medical ward, OPD and surgical ward. The red bars correspond to isolates originating from blood; the black bars correspond to isolates originating from all other specimen types. Third row: the distribution of PCR-confirmed Outbreak Lineage 1 strains containing *bla*_NDM__-1_. Arrows signify the first and last occasions on which *bla*_NDM__-1_ positive blood-borne *Klebsiella pneumoniae* Outbreak Lineage 1 strains were isolated.

The 31 ST15 isolates predominated and were highly temporally associated; therefore, we constructed a refined maximum likelihood phylogeny to study these isolates in greater detail. The phylogeny emphasised a highly structured clonal population composed of 25/31 isolates (14 from blood), which we designated Outbreak Lineage 1. A core genome alignment of these strains revealed a total of 66 non-recombinant SNPs among 24 strains (one strain was excluded due to extensive recombination). Notably, the Outbreak Lineage 1 isolates originating from the adult intensive care unit (AICU) and the PICU fell into distinct clades, as did the NICU and nursery isolates—despite their apparent proximity in time and locality (Fig2A). These data predict that all of these cases were unlikely to represent an on-going transmission chain of the NICU-based strains outside of the NICU. Nevertheless, an additional strain within the Outbreak Lineage 1 NICU cluster was isolated from an individual in the outpatient department more than 3 months after the first NICU outbreak subsided (Fig2A).

The genome sequencing data showed that ST1559 isolates were highly clonal and exhibited a lesser degree of phylogenetic diversity than the ST15 isolates, harbouring only 42 SNPs across the core genome of the 19 sequenced isolates. The cases attributable to ST1559 arose later than Outbreak Lineage 1 (between September and December 2012); therefore, we named this group Outbreak Lineage 2.

### Assessing the temporal ranges of the outbreak lineages

The 24 Outbreak Lineage 1 strains mainly originated from a range of patient samples in the NICU complex in May and June 2012, which is consistent with the timing of the first peak in *K. pneumoniae* isolations in the microbiology department. To infer the temporal scope of the Outbreak Lineage 1 strains, we estimated the time of the most recent common ancestor (TMRCA) of sequenced Outbreak Lineage 1 isolates using Bayesian phylogenetic methods implemented in BEAST (Fig2A) (Drummond & Rambaut, 2007). We estimated a median substitution rate of 2.7 × 10^−6^ substitutions per site per year for Outbreak Lineage 1 (95% highest posterior density (HPD): 6.8 × 10^−7^–5.0 × 10^−6^), corresponding to an inferred MRCA dating back to early 2011 (95% highest posterior density interval [HPD] 2009–2012) (Fig2B). However, we interpreted these data with caution noting a relatively weak correlation (*R*^2^ = 0.134) between root-to-tip branch lengths and the time of isolation, which was consistent with the outbreak occurring over a short time frame.

**Figure 3:**
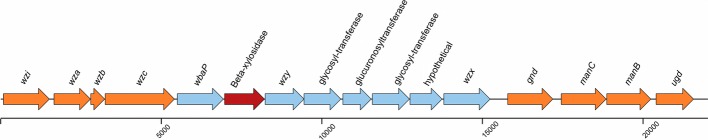
The architecture of the novel capsule biosynthesis cluster (*cps*) in the ST15 Outbreak Lineage 1 *Klebsiella pneumoniae* Graphical representation showing the composition of the *cps* gene cluster within the Outbreak Lineage 1 *K. pneumoniae* isolates. The genes are identified by either their common name (e.g. *wba*P) or their predicted function (e.g. beta-xylosidase). Genes that are shared by other strains of *K. pneumoniae* are coloured orange, and genes specific to this cps cluster are coloured blue and red. The beta-xylosidase gene (red) was selected as the target for lineage-specific PCR amplification.

To address the limitations of the Bayesian phylogenetic analysis, we screened an additional 412-stored *K. pneumoniae* isolated in the hospital microbiology laboratory between 2005 and 2013 by PCR amplification. To achieve this, we used comparative genomics on a WGS scale to identify unique regions found in the 25 Outbreak Lineage 1 strains, which included a chromosomal cluster specific for its novel capsule type (Fig3), as well as a marker for a lineage-specific (pNDM-MAR like) plasmid (see below for details of these regions) (Supplementary Table S2). We additionally included primers for the *bla*_NDM-1_ cassette, which was present in 19 of the 25 Outbreak Lineage 1 strains and absent by an apparent perfect excision from pNDM-MAR in the remaining six isolates. By PCR amplification using the specific novel capsular type and pNDM-MAR primers, we identified a further 46 suspect Outbreak Lineage 1 isolates, of which 35 additionally harboured *bla*_NDM-1_. These supplemental isolates were largely confined to the NICU, PICU and the nearby nurseries, with the first confirmed isolation dating back to September 2011 (Fig2C). Despite the limitations of the Bayesian phylogenetic reconstruction, the complementary PCR screening data suggest that Outbreak Lineage 1 was circulating within these wards for at least 6 months prior to recognition of the outbreak and predicted that the lineage likely arose within the hospital in early 2011.

Again, using the WGS data, we identified a similarly informative region within the genome of the Outbreak Lineage 2 isolates to study the pattern of spread of this lineage across the hospital. Resulting PCR amplifications of the Outbreak Lineage 2**-**specific capsule gene (K14) [see below for details of this region (Table1)] confirmed that there were likely at least 26 infections caused by this strain (14 cases of bacteraemia) on the NICU, PICU, nurseries and medical wards. A strain belonging to this Outbreak Lineage was first isolated in a medical ward in August 2012, and another was isolated in December after the suspected outbreak in November.

**Table 1:** The antimicrobial resistance and hypothetical virulence determinants in *Klebsiella pneumoniae* Outbreak Lineages 1 and 2

Organism	Location	Genetic element	Function	Presence in *K. pneumoniae* strains
ST15 Outbreak Lineage I	pNDM-MAR like	*bla*_*NDM-1*_*/qnrB1/bla*_*CTX-M-15*_*/catB3/OXA-30/acc(6’)-lb-cr*	carbapenem/fluoroquinolone/cephalosporin, aztreonam/chloramphenicol/oxacillin/aminoglycoside resistance	Outbreak 1 only
	Class I integron of pCTX-M3	*arm*A/*mrs*E-*mph*E/*sul*1/*dhf*r/*aad*A2	Aminoglycoside/macrolide/sulphonamides/trimethoprim/spectinomycin resistance	Outbreak 1 only
	Tn5393c of *Aeromonas* pRAS2	*str*A-*str*B	Streptomycin resistance	Outbreak 1 only
	pKN-LS6 plasmid family	*cus/sil/ars* locus	Copper/silver/arsenite ions resistance	Outbreak 1 only
	Chromosome	Novel capsular biosynthesis cluster (K-type)	Immune escape	Outbreak 1 & 3 other ST15
	Chromosome	Filamentous hemagglutination adhesin locus (ShlB/FhaC/HecB)	Hypothetical adhesins	Outbreak 1 & 5 other ST15
	Chromosome	*K. pneumoniae* KTCC 2242 phage mediated fimbrial cluster	Fimbrial biosynthesis	All ST15
	Chromosome	*K. pneumoniae* 342 T6SS	Host–pathogen interaction and interbacterial communication	All ST15 & all ST14
	Chromosome	ICE-KpNp1	Metal ion uptake systems, yersiniabactin and mobilisation proteins	Outbreak 1 only
	Chromosome	KR116 KpGI-5 genomic island	Biofilm formation	All ST15
	Chromosome	*gyr*A mutation S83F, D87A	Fluoroquinolone resistance	All ST15 + 1 background lineage
	Chromosome	*par*C mutation S80I	Fluoroquinolone resistance	All ST15 + 11 background lineages
ST1559 Outbreak Lineage 2	pKDO1 plasmid family	*sul*2/*str*A-*str*B/TEM-1/CTX-M-15	Sulphanomides/streptomycin/ampicillin/cephalosporins, aztreonam resistance	Found in multiple lineages
	Undetermined plasmid	OXA-30/aac(6’)-lb-cr	Oxacillin/aminoglycoside resistance	Found in multiple lineages
	Undetermined plasmid	*qnr*B1	Fluoroquinolone resistance	Found in multiple lineages
	IS5 mediated, undetermined plasmid	*C. sakazakii* thermoresistance cluster	Hypothetical heat shock resistance	Outbreak 2 only
	pKPN_CZ backbone	*cus/sil* locus	Copper/silver resistance	Outbreak 2 & 4 background lineages
	pCC416	*ars* locus	Arsenite resistance	Outbreak 2 & 3 background lineages
	Undetermined	Extra *ast*ABCDE	Amino acid transport and metabolism	Outbreak 2 only
	Undetermined	IS903 flanked transposome	Amino acid transport and metabolism	Outbreak 2 only
	Chromosome	Yersiniabactin and T4SS	Iron uptake and mobilisation protein	Outbreak 2 only
	Chromosome	*mrk*ABCD locus	Biofilm formation	Outbreak 2 & 4 background lineages

### Comparative genomics and lineage-specific gene content

Aiming to understand the genetic basis for the disease phenotypes associated with Outbreak Lineage 1 and Lineage 2, we performed comparative genome analyses by aligning the entire gene contents of the two outbreak lineage strains against the background (non-outbreak) hospital strains. Genetic features that were unique to either outbreak lineage and/or exhibiting potential virulence or antimicrobial resistance were considered as relevant disease markers.

We were able to differentiate the isolates belonging to Outbreak Lineage 1 from all others circulating in this hospital because they possessed a region encoding a previously undescribed capsular antigen biosynthesis (*cps*) cluster which constituted an uncharacterised *K. pneumoniae* capsular K-type (Fig3). This lineage also possessed a wide complement of additional elements related to virulence and persistence in *Klebsiella* (Podschun & Ullmann, 1998; Schroll *et al*, 2010) (Table1). While type 1 fimbrial cluster was found in all *K. pneumoniae* strains, this lineage harboured several additional fimbrial and adhesin gene clusters, including a distinctive locus II type six secretion system (T6SS) similar to that found in *K. pneumoniae* 342 (> 99% identity) (Sarris *et al*, 2011) and an intact copy of a genomic island (KR116 KpGI-5) containing a fim2 biofilm associated gene cluster (Van Aartsen *et al*, 2012) (Table1). Iron acquisition has been strongly implicated in virulence in *K. pneumoniae* (Luck *et al*, 2001; Raymond *et al*, 2003; Ma *et al*, 2005; Lawlor *et al*, 2007). Besides the conventional enterobactin that was found in all strains, Outbreak Lineage 1 again carried a surplus of iron-acquiring systems, including *kfu*,*fec* and yersiniabactin. The yersiniabactin encoding region was identical across Outbreak Lineage 1, distinct from that of the Outbreak Lineage 2 isolates, and appeared to have been co-transferred with additional features as described in other members of the Integrative Conjugative Element (ICE) family (Paauw *et al*, 2010).

We additionally identified several loci associated with persistence and pathogenesis in the Outbreak Lineage 2 strains (Table1). Of note, we identified multiple loci associated with attachment, including a biofilm encoding *mrk*ABCD type 3 fimbriae locus (Schroll *et al*, 2010) (Table1). These Outbreak Lineage 2 strains were defined by gene alignment to be capsule type K14. The Outbreak Lineage 2 isolates carried a plasmid-encoded *fec* locus, enterobactin and a yersiniabactin operon. We additionally identified a thermal resistance (temperature survival) gene cluster exhibiting extensive DNA homology to an equivalent region described in *Cronobacter sakazakii* (Gajdosova *et al*, 2011).

### Antimicrobial resistance gene content of the Outbreak Lineages

**Figure 4:**
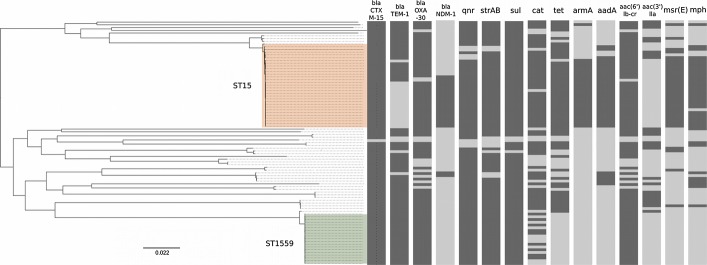
The resistome of the 89 hospital isolates of *Klebsiella pneumoniae* Maximum likelihood phylogeny based on core genome SNPs of the 89 *Klebsiella pneumoniae* isolates adapted from Fig1 set against the resistome of 15 antimicrobial resistance genes (named at the top of the figure). The dark and light grey boxes show the presence and absence of the specific resistance genes in the corresponding isolates, respectively. *bla*, beta-lactamase; *qnr*,*aac(6’)-Ib-cr*, quinolone resistance genes; *strAB*, streptomycin resistance genes; *sul*, sulphonamide resistance gene; *cat*, chloramphenicol resistance gene; *tet*, tetracycline resistance gene; *armA*,*aadA*,*aac(3)-IIa*, aminoglycoside resistance genes; and *msr(E), mph*, macrolide resistance genes.

Apart from the virulence-associated genes, we additionally hypothesised that the apparent success of *K. pneumoniae* Outbreak Lineage 1 was further driven by selection induced by antimicrobial usage within the hospital. All Outbreak Lineage 1 isolates were resistant to a broad array of antimicrobials (Fig1C, Supplementary Table S1). Using the WGS data, we predicted all of the known resistance genes (resistome) found within the sequences (Table1 and Fig4). Consistently, the resistome of Outbreak Lineage 1 was extensive and distinct from the other *K. pneumoniae* strains, predicting generic susceptibility to colistin and tigecycline only. All of the Outbreak Lineage 1 organisms harboured an MDR plasmid, which shared a high degree of DNA homology (> 99% identity) to the previously described plasmid pNDM-MAR (Villa *et al*, 2012). Within Outbreak Lineage 1, the presence of *bla*_NDM-1_ correlated precisely with resistance to carbapenems in isolates originating from the NICU (*n* = 13), the AICU (*n* = 2), medical ward (*n* = 1) and the outpatient department (*n* = 1). Furthermore, the Outbreak Lineage 1 isolates were found to harbour a pCTX-M3 class I integron encoding resistance to an assortment of antimicrobials including aminoglycosides (*armA*) and macrolides (*mrsE* and *mphE*) (Table1 and Fig3). Correspondingly, all of the Outbreak Lineage 1 strains also possessed mutations in *gyr*A (S83F, D87A) and *par*C (S80I), which together with *qnrB1* and *aac(6’)-lb-cr* reduce susceptibility to fluoroquinolones (Hopkins *et al*, 2005). We additionally noted some further ward-specific diversification of Outbreak Lineage 1, with some of the NICU strains having mutations in the *phoP*, of which the impact on polymyxin susceptibility is not yet well understood (Fig2A). Further, the AICU and PICU Outbreak Lineage I strains gained a pFOS-HK plasmid containing the macrolide resistance genes *erm*A/B (Fig2A). The Outbreak Lineage 2 strains exhibited less antimicrobial resistance than the Outbreak Lineage 1 strains and were generically more similar to the background hospital isolates; this was reflected in the antimicrobial resistance gene content. The Outbreak Lineage 2 strains harboured three beta-lactamases (*bla*_CTXM-15_, *bla*_TEM-1_ and *bla*_OXA30_), the fluoroquinolone resistance genes *qnrB1* and *aac(6’)-lb-cr* and variable presence of *cat* (chloramphenicol acetyltransferase).

## Discussion

Here, we have presented an investigation of two outbreaks of MDR *K. pneumoniae* infections in adjoining high-dependency paediatric units in a hospital in Nepal. The outbreaks were characterised by a high proportion of bloodstream infections and a high case-fatality rate. We used WGS in an attempt to characterise strain circulation and diversity in this hospital, and comparative genomics to identify genetic traits that may be associated with the disease phenotype and the persistence of these strains. The notable advances highlighted in this study, in comparison to previous investigations of hospital outbreaks of *K. pneumoniae,* are the use of the large-scale WGS data to deduce the bacterial isolates involved in the outbreaks and to use these data to retrospectively define the scope of outbreak strains across the hospital over a protracted period. These approaches have permitted us to interrogate these serious hospital outbreaks of *K. pneumoniae* with a degree of resolution that would be inaccessible through conventional methods. The WGS approach has previously been implemented to study hospital outbreaks of organisms such as KPC-producing *K. pneumoniae* in the U.S. (Snitkin *et al*, 2012) and *Staphylococcus aureus* in the U.K. (Harris *et al*, 2013). We think that this study contributes major insights into the epidemiology of *K. pneumoniae*: a major global, and often overlooked, Gram-negative pathogen. Further, this investigation was performed in a resource-limited hospital setting, an example of an environment where hospital outbreaks of *K. pneumoniae* can be associated with high mortality.

*Klebsiella pneumoniae* is a common community and hospital-acquired pathogen, yet is especially prevalent and vicious in high-risk patients in low-income countries (Escobar Pérez *et al*, 2013; Espedido *et al*, 2013; Poirel *et al*, 2014). As such, *K. pneumoniae* are commonly associated with poor outcomes through respiratory tract infections and bloodstream infections in these locations. This is partly because *K. pneumoniae* are particularly successful in adapting to specific niches through the acquisition of genetic material via horizontal gene transfer (HGT) (Ma *et al*, 2005; Lawlor *et al*, 2007; Fouts *et al*, 2008) and are an extensive reservoir of antimicrobial resistance genes within healthcare settings (Tada *et al*, 2013; Wang *et al*, 2013). The organisms studied here were no exception, with 100% and 65% of the 2012 collection of 270 *K. pneumoniae* exhibiting resistance to cefotaxime and ofloxacin, respectively. Further, a resistome analysis of the 89 sequenced *K. pneumoniae* revealed an exhaustive repertoire of antimicrobial resistance genes, thus illustrating the threat that circulating antimicrobial resistant *K. pneumoniae* pose in this hospital (Basnyat, 2014). Indeed, the acquisition of a carbapenemase in an already MDR background appears to have directly contributed to one of the two identified outbreaks.

Our analysis of the WGS data permitted us to identify two discrete *K. pneumoniae* lineages causing two temporally distinct outbreaks of bacteraemia in the high-dependency paediatric wards, named Outbreak Lineage 1 and 2, respectively. The first *K. pneumoniae* outbreak was the largest and the most devastating, causing a minimum of 34 bloodstream infections, of which 28 were fatal. *K. pneumoniae* Outbreak Lineage 1 was an ST15 strain, in which the majority of isolates harboured the carbapenemase gene *bla*_NDM-1_, which appears to have become well adapted for sustained transmission within this hospital environment, as evidenced by its prolonged detection prior to the outbreak in 2011 through to the latter part of 2013. The distribution of Outbreak Lineage 1 in the hospital, along with its ward-structured phylogeny, supports a scenario where, following initial introduction into adjacent wards, these strains have undergone a series of independent and highly localised micro-clonal expansions. We speculate that the enhanced virulence potential observed in this outbreak clade was induced by the cumulative introductions of several mobile genetic elements (MGEs). The discrepancy in the scale of the accessory genome between the Outbreak Lineage 1 strains and the non-Outbreak Lineage 1 strains implies that the progenitors of this Outbreak Lineage had access to a wider gene pool than that of other hospital *K. pneumoniae* strains, or alternatively that this strain is particularly receptive to HGT. Indeed, the fixation of the novel capsular antigen predates the diversification of Outbreak Lineage 1 (as evidenced by three novel K-type strains without pNDM-MAR). This novel capsule type may have expedited the initial phase of strain persistence in the hospital through an enhanced capacity for attachment and/or colonisation. The acquisition of the yersiniabactin operon and the pNDM-MAR plasmid were unquestionably pivotal events in the emergence of this strain within the hospital. The active gene acquisition and diversification of some Outbreak Lineage 1 strains during the period of investigation, as exemplified by gain of a predicted novel plasmid and the macrolide resistance (*erm*B) cassette in the AICU and PICU clusters, further supports our hypothesis that this lineage was particularly receptive to genetic material encoding additional phenotypes.

The second *K. pneumoniae* outbreak occurred later in 2012 and, whilst still major, was less severe (with respect to scale) than the first outbreak, causing 26 infections (14 bacteraemia cases with eight fatalities). The Outbreak Lineage 2 strains were also MDR, yet all strains were susceptible to carbapenems and aminoglycosides. However, like Outbreak Lineage 1, Outbreak Lineage 2 also had an expanded accessory genome containing several iron-acquiring systems, including a yersiniabactin distinct to those found in Outbreak Lineage 1. Crucially, siderophores such as yersiniabactin serve the key function of iron acquisition for *K. pneumoniae* in low iron conditions, and yersiniabactin is crucial for replication in the host (Lawlor *et al*, 2007; Bachman *et al*, 2011). We speculate that the excess of these iron acquisition systems contributed greatly to the ability of both outbreak lineages of *K. pneumoniae* to cause systemic infections, potentially by allowing more efficient replication whilst in the bloodstream through enhanced iron chelation.

The endemicity of NDM-1 in South Asia is being facilitated by efficient HGT between differing bacterial species, as exquisitely demonstrated by the presence/absence of *bla*_NDM-1_ in Outbreak Lineage 1 and on differing plasmids of other STs in this hospital (Fig1C). The data suggest that *bla*_NDM-1_ is common in healthcare facilities in Nepal. Indeed, whilst this work was in progress, NDM-1-producing *K. pneumoniae* was identified in another major hospital in the Kathmandu Valley (Tada *et al*, 2013). It appears that the strain identified in this study shares some of the traits of Outbreak Lineage 1. Both the strains from the cited study and Outbreak Lineage 1 were of CC14 (specific ST not stated) and both possessed a pCTX-M3 *arm*A integron cassette, *bla*_NDM-1_, and had identical fluoroquinolone resistance inducing mutations (S83F, D87A in *gyr*A and S80I in *par*C). Whilst the genetic environment of *bla*_NDM-1_ was not specified in the previous study, it is possible that these two clades/clones are closely related. We suspect that these strains have covertly spread throughout a number of healthcare facilities in the region; detailed and broader follow-up work and phylogenetic reconstruction are clearly warranted to understand the true extent of the temporal and geographical circulation of these strains in Nepal.

Our work contains some limitations that should be considered in the context of this investigation. Firstly, the strains subjected to WGS were collected over a relatively short time frame, with our analysis concentrated on strains falling within the two outbreak peaks. This approach restricted our investigative power over a more extensive timescale. Bayesian phylogenetic inference suggests that Outbreak Lineage 1 likely emerged in 2011. This date was consistent with our clade-specific PCR amplification results; however, the timescale of sampling and rapidity of these outbreaks offer only limited variation across the core genome with which to infer evolutionary and epidemiological processes, and thus may impact the robustness of this analysis. Further, we did not exhaustively perform WGS on all *K. pneumoniae* isolated in the hospital in 2012. However, we argue that our strain selection and supplementary PCR analysis were sufficient to estimate the extent of the outbreaks. Of course, we cannot guarantee that all outbreak strains inferred by PCR amplification were closely related, as detection of the selected loci may be compromised by HGT within the circulating Gram-negative population. However, these capsular and antimicrobial resistance genes were relevant in this location, and such targets have been previously exploited for a similar purpose (Villa *et al*, 2012; Chen *et al*, 2014). Since the time frame for this study was short, we predict that the impact of capsule misidentification due to HGT in the two highlighted lineages was minimal.

Using an approach similar to the one described here, the genomic diversity and spread of KPC-producing ST258 *K. pneumoniae* in hospitals in the U.S. has been recently investigated (Deleo *et al*, 2014; Wright *et al*, 2014). Further, a *cps* gene cluster introduction into these ST258 strains has been shown to facilitate substantial divergence within this ST, and retrospective PCR based on distinct capsular markers has been utilised to probe its expansion across U.S. healthcare facilities (Chen *et al*, 2014). While phylogenetics and comparative genomics have been performed previously for outbreaks of *K. pneumoniae*, such studies have not typically considered the endemic background strain diversity and have focussed on specific problematic lineages in developed countries (Snitkin *et al*, 2012; Ramos *et al*, 2014). Our work is novel in its approach to utilising genomic data to explore highly fatal nosocomial outbreaks of *K. pneumonia*e in a low-income setting. Most significantly, we identified the emergence of a capsule switching NDM-1-bearing ST15 strain in a single hospital. NDM-1-producing ST15 *K. pneumoniae* has been reported repeatedly (Poirel *et al*, 2011; Arpin *et al*, 2012; Hu *et al*, 2013), but its transmission ability and pathogenic capacity have not been assessed. We propose that further studies should concentrate on the diversity and spread of this specific clone, particularly in healthcare settings in developing countries.

In conclusion, we have spatiotemporally characterised invasive *K. pneumoniae* populations in one hospital in Nepal using WGS. These data permitted a comprehensive understanding of the emergence of two highly virulent clones, which could then be retrospectively identified using relevant lineage-specific genetic determinants. Owing to the severity and the mortality of these outbreaks, it is regrettable that some form of routine genetic characterisation was not performed in real time. After the first cases of *K. pneumoniae* bacteraemia, NDM-1 PCR amplification and MLST would have added some vital insights into these outbreaks in a clinically relevant time frame and may have reduced their magnitude. We suggest that the early detection of a specific NDM-1 clone on the NICU complex in 2011 would have alerted hospital staff to intervene, permitted on-going surveillance with a review of the antimicrobial treatment policy and sustained screening and patient isolation. The added benefit of WGS was to dissect these outbreaks in extremely fine detail. We argue that some form of real-time genetic characterisation, and clade-specific PCR amplification, should undoubtedly factor into future routine microbiology in such hospitals to rapidly provide data required to intervene in such outbreaks in a timely manner.

## Materials and Methods

### Ethics statement

The institutional review boards of the Patan Academy of Health Sciences (PHAS) and the Nepal Health Research Council (NHRC) provided ethical approval for this study.

### Setting

Patan Hospital is a 450-bed government hospital providing emergency and elective outpatient and inpatient services located within the Kathmandu Valley in the Sub-Metropolitan City of Lalitpur, Nepal. This study describes a *K. pneumoniae* outbreak in this hospital in 2012. The outbreak appeared to be restricted to the three nurseries (A, B and C), the NICU and the PICU on the first floor and the medical ward and the adult intensive care unit (AICU) directly above on the second floor (as described in Fig1A). The NICU is a seven-bed ward (one bed for isolation) for neonates (≤ 28 days old) requiring intensive therapy. The ward typically treats babies with intrauterine growth restriction (IUGR) or premature neonates with sepsis or requiring ventilation. It has seventeen full-time medical staff (eight nurses and nine doctors) with four staff on shift at any one time. The PICU is a six-bed ward for children > 28 days old requiring intensive therapy. The ward typically treats children with shock, most often due to sepsis and those requiring mechanical ventilation. The PICU has nineteen full-time medical staff (ten nurses and nine doctors). The nurseries are adjacent to the maternity ward and the NICU and PICU, and newborn children are routinely transferred into them for general observations. Combined, these nurseries contain twenty beds. The doctors regularly move between the NICU, PICU and nurseries, but the nurses are restricted to their respective wards. The second floor AICU is an eight-bed ward for adults (> 18 years) requiring intensive therapy, typically those presenting with shock, cardiac emergencies and those requiring mechanical ventilation. The AICU has eight full-time medical staff (four nurses and four doctors) and is located adjacent to a general medical ward containing sixty beds (two for isolation) for patients with a variety of conditions. Data relating to individual patient treatment regimes and clinical variables (other than death) were not accessible.

### Bacteriology and antimicrobial susceptibility testing

The isolates described in this study were obtained from cultures of clinical specimens taken as part of routine clinical care from Patan Hospital inpatients between May and December 2012. Micro-organisms were isolated and identified using routine microbiological methods. Briefly, blood cultures were processed using the BD BACTEC™ (Becton Dickinson, UK) Instrumented Culture System incubating BACTEC™ Peds Plus™/F culture vials containing 1–2 ml of peripheral blood for children. Adult blood samples (typically > 5 ml) were collected in BACTEC™ Plus Aerobic/F Culture Vials. Samples flagged as positive by the BD BACTEC™ system were sub-cultured onto MacConkey and blood agar plates and incubated at 37°C to isolate the infecting organism. All other clinical specimens (e.g. tracheal aspirates, wound swabs, urine) were processed according to standard microbiological procedures. *K. pneumoniae* (and other Gram-negative organisms) was identified using standard biochemical tests and API20E testing, following the manufacturer's instructions (Bio Merieux, France).

Antimicrobial susceptibilities were tested at the time of isolation by the modified Bauer-Kirby disc diffusion method, as recommended by the CLSI guidelines (CLSI, 2012). Mueller-Hinton agar and antimicrobial discs were purchased from Unipath, Basingstoke, United Kingdom. *Escherichia coli* ATCC 25922 was used as the control strain. The inhibitory zone sizes were recorded and interpreted according to current CLSI breakpoint guidelines (CLSI, 2012). The following antimicrobials were used for *K. pneumoniae* susceptibility testing: amoxicillin (AMX), cefotaxime (CTX), ciprofloxacin (CIP), ofloxacin (OFX), trimethoprim/sulfamethoxazole (SXT), gentamicin (GEN), amikacin (AMK), chloramphenicol (CHL), meropenem (MEM) and imipenem (IPM).

### Whole-genome sequencing

We selected a random subset of 90 *K. pneumoniae* (using a random integer generator) from a collection of 135 *K. pneumoniae* that were isolated and stored in Patan Hospital between May and December 2012. This collection contained *K. pneumoniae* isolated from blood (*n* = 28), urine (*n* = 27), catheter and CV tips (*n* = 32), respiratory tract infections (*n* = 20), pus (*n* = 14) and other infection sites (*n* = 14) (selected isolated outlined in Supplementary Table S1). These organisms were subjected to DNA extraction by using the Wizard Genomic DNA Extraction Kit (Promega, Wisconsin, USA), and 2 μg was subjected to whole-genome sequencing (WGS) on an Illumina HiSeq2000 Platform (Illumina, San Diego, USA) to generate 150 bp paired-end reads, as previously described (Quail *et al*, 2008). Reads were mapped to the reference *K. pneumoniae* MGH78578 by using BWA, and QC stats were exported to graphs and manually assessed (Li & Durbin, 2010). The average Phred quality score of the all sequencing read sets was 35 (33.7–37). *De novo* assemblies were generated for each read set using Velvet (version 1.2.03) and VelvetOptimiser, with each read set mapped back to each assembly (Zerbino & Birney, 2008). The resulting *K. pneumoniae* short-read sequence data were deposited in the European Read Archive under the Accession Numbers ERR349747 to ERR349855 inclusive. The assemblies of representatives of two outbreak lineages are available at GENBANK under Accession numbers CDQG01000001-CDQG01000097 (Outbreak Lineage 1) and CDPE01000001-CDPE01000062 (Outbreak Lineage 2), and the novel capsule biosynthesis gene from Outbreak Lineage 1 is available under the Accession Number LN714331.

### Genome annotation, core genome construction andphylogenetic analysis

For each *de novo* assembly, coding sequences were predicted and annotated using the rapid prokaryotic genome annotation tool, Prokka (Seemann, 2014). Predicted coding sequences comprising more than 5% undetermined nucleotides or shorter than 120 nucleotides in length were excluded from further analysis. Coding sequences were translated into proteins, and protein clustering was performed iteratively by CD-hit (Li & Godzik, 2006) initially with 100% identity and 100% length match, then reducing by 0.1% until reaching a minimum 98% identity. The “core” *K. pneumoniae* genome was defined as concatenation of coding sequences present in one copy in all of the final 89 (one failure) *K. pneumoniae* assemblies. We used this core genome alignment to construct a maximum likelihood phylogeny of the 89 *K. pneumoniae* isolates using RAxML after removing predicted regions of recombination by using Gubbins (Stamatakis, 2006; Croucher *et al*, 2014). In brief, Gubbins predicts and removes recombinant regions using a spatial scanning statistic to identify genomic regions of significantly raised SNP density on each branch of the tree. Once recombinant regions are removed, the tree is recalculated and the process repeated until the tree or recombination predictions are the same as a previous iteration or when a user-specified maximum number of iterations are reached. Specific loci of interest were retrieved from the annotated assemblies, aligned with MUSCLE (Edgar, 2004) and visualised in SeaView (Gouy *et al*, 2010). Multilocus sequence typing (MLST) was performed for all *K. pneumoniae* isolates by extracting the seven typing genes *in silico* (*gapA*,*infB*,*mdh*,*pgi, phoE*,*rpoB*,*tonB*) (Diancourt *et al*, 2005). Capsule genotyping was performed by comparing the sequence of the *wzc* gene to those deposited in Genbank (Pan *et al*, 2013). The resistance gene profiles and content (resistome) of each isolate were characterised using a manually curated database, based on the ResFinder database (Zankari *et al*, 2012). Each gene in the database was mapped against the isolate assemblies to identify complete genes. Where fragments matching the 5′ or 3′ ends of resistance genes were identified at contig boundaries, sequencing reads were mapped to all matching candidate genes and their presence assessed based on mapping coverage across the gene.

### Accessory genome assembly

To improve the assembly of accessory regions, sequencing reads for each isolate were mapped against the *K. pneumoniae* 1084 reference genome using SMALT (https://www.sanger.ac.uk/resources/software/smalt/) (Lin *et al*, 2012). Unmapped read pairs were extracted and assembled as described above. Contigs were discarded if they were shorter than 1,000 bp or contained a region of at least 1,000 bp that matched the reference genome using BLAST. The remaining contigs from all isolates were compared in a pairwise manner using BLAST. In each comparison, contigs were retained if they contained at least 1,000 bp of unmatched sequence, or if they were the longer of the two compared sequences. When two or more contigs were identical, only one was retained and all other contigs were discarded. The presence or absence of contigs in the remaining accessory genome was assessed for each isolate by mapping sequencing reads against the reference genome and accessory contigs simultaneously.

### Comparative genomics

The pan-genome of 89 *K. pneumoniae* strains constructed by the accessory genome assembly method described above was used for comparative genomic analysis to identify genetic features distinct to the two outbreak lineages (present in either outbreak lineage but absent in almost all remaining strains). These identified genetic features were individually assessed using BLAST to determine their origin, location within the genome (plasmid or chromosomal borne) and function based on homology to the highest score BLAST hits. Poorly characterised, putative prophages and functionally unknown regions were excluded, and regions for which annotations matched with known potential virulence factors were retained for further analysis.

### Bayesian phylogenetic methods

To investigate the timescale of the emergence and spread of these *K. pneumoniae* outbreaks, we inferred a maximum likelihood phylogeny for 24 *K. pneumoniae* isolates which represented the primary outbreak strain (described herein as Outbreak Lineage 1) using PhyML under the HKY85 + Γ_4_ model of substitution (Guindon *et al*, 2010). To test for temporal signal in the phylogeny, we assessed the correlation between root-to-tip distance and the date of isolation (day, month, year) in Path-O-Gen (v1.4). A Bayesian Markov chain Monte Carlo (MCMC) approach was then applied in the BEAST package (v1.8) to infer the posterior distribution of trees and determine dates of divergence of lineages of interest (Drummond & Rambaut, 2007). We used an HKY85 + Γ_4_ substitution model, a relaxed lognormal molecular clock, a constant size demographic model and tip dates defined as day, month and year of isolation; Bayes factor testing determined this model to be the best fit of various models tested. Analyses were run in triplicate using a continuous 200 million generation MCMC chain with samples taken every 20,000 generations. All analyses were run until parameters reached convergence with ESS > 200, as assessed using Tracer (v1.5). Triplicate runs were combined using LogCombiner (v1.8) with removal of 20% for burn-in.

### Retrospective PCR screening

To retrospectively screen for organisms belong to Outbreak Lineage 1 and Outbreak Lineage 2, we identified *K. pneumoniae* lineage-specific regions using comparative genomics, as described above. Primers were designed (or selected from previous publications) for lineage-specific genes in the capsule regions, the *bla*_NDM-1_ cassette and the corresponding MDR plasmid. These primers, outlined in Supplementary Table S2, were combined to form a multiplex with predicted amplification sizes of 400, 600, 1,000 and 1,300 bp. PCR amplification was performed on the 412 *K. pneumoniae* that were isolated and stored in Patan Hospital between 2005 and 2013. DNA was extracted by inoculating the cultured *K. pneumoniae* into 50 μl of sterile water and exposing them to 100°C using a microtube heating block (Stuart equipment, UK) for 5 min. Extractions were centrifuged briefly and 2 μl of supernatant was subjected to multiplex PCR amplification for 5 min at 94°C, 35 cycles of 30 s at 94°C, 30 s at 55°C and 105 s at 72°C, followed by 7 min at 72°C. PCR was performed using FastStart Taq DNA polymerase (Roche, Switzerland) and icycler thermal cycler (Bio-Rad, USA). PCR amplifications were UV visualised after agarose gel electrophoresis and staining with ethidium bromide.

**The paper explained**

**Problem**

*Klebsiella pneumoniae* is a Gram-negative bacterium that colonises humans and animals. The organism is adept at gaining multiple antimicrobial resistance genes and, given the right opportunities, can cause serious infections in susceptible individuals. In 2012 there was an outbreak of highly drug resistant *Klebsiella pneumoniae* bloodstream infections on high-dependency wards for infants in our hospital in Kathmandu, Nepal. This outbreak had two peaks within the year and a mortality of 75%.

**Results**

We selected many of these outbreak strains and some of non-outbreak *Klebsiella pneumoniae* from across the hospital and performed whole-genome sequencing. Our analysis revealed two independent outbreak strains of *Klebsiella pneumoniae* that we named Outbreak Lineage 1 and Outbreak Lineage 2. We were able to use these data to reconstruct these outbreaks through time and across the affected wards with meticulous accuracy. We found that strains belonging to Outbreak Lineage 1 were circulating in the hospital for 6 months before the outbreak and had gained genes facilitating infection and antimicrobial resistance.

**Impact**

This work allows a comprehensive insight into how hospital outbreaks of drug resistant *Klebsiella pneumoniae* occur in low-income settings. The power of these data outlines that some real-time genetic characterization should be performed during future healthcare infection control practices in both high- and low-income settings to identify outbreaks and limit onwards transmission.

## For more information

http://www.cdc.gov/drugresistance/threat-report-2013/pdf/ar-threats-2013-508.pdf

https://www.gov.uk/government/uploads/system/uploads/attachment_data/file/244058/20130902_UK_5_year_AMR_strategy.pdf

http://www.pasteur.fr/recherche/genopole/PF8/mlst/primers_Kpneumoniae.html

https://www.sanger.ac.uk/resources/software/

http://www.oucru.org/enterics/

http://www.sciencedaily.com/releases/2008/12/081201105706.htm

## Supporting Information

Supplementary Tables

Review Process File
