# Supplementary material for: A high-resolution genomic analysis of multidrug-resistant hospital outbreaks of Klebsiella pneumoniae
Source: EMBO Mol Med. 2015 Feb 20;7(3):227–39. doi: 10.15252/emmm.201404767 (PMC4364942; doi:10.15252/emmm.201404767)
Supplement: Supplementary file 1 [file emmm0007-0227-sd1.pdf]

Supplementary information

Contents

Table S1. The 90 bacterial isolates selected for whole genome sequencing in this study

Table S2. The primers used for retrospective PCR detection of Outbreak Lineages 1 and 2

Supplementary references

Table S1. The 90 bacterial isolates selected for whole genome sequencing in this study

ID	Orgasism	Date of isolation	Ward of isolation	Specimen	Sequence type (ST)	Capsule type	MEM	IMP	CTX	CIP	OFX	GEN	AMK	CHL	STX
10315_6#1	*Klebsiella pneumoniae*	16/5/2012	Medical	Urine	37	K12	S	S	R	R	S	S	S	NR	S
10315_6#10	*Klebsiella pneumoniae*	16/6/2012	Nursery C	Blood	14	K2	S	S	R	R	S	R	S	S	R
10315_6#11	*Klebsiella pneumoniae*	17/6/2012	ICU	Tracheal aspiration	15	novel Ktype	R	R	R	R	R	R	R	R	R
10315_6#12	*Klebsiella pneumoniae*	19/6/2012	ICU	Blood	307	NK29 (unidentified K type)	S	S	R	R	R	R	S	S	R
10315_6#13	*Klebsiella pneumoniae*	19/6/2012	ICU	Sputum	15	novel Ktype	S	S	R	R	R	R	R	R	R
10315_6#17	*Klebsiella pneumoniae*	26/6/2012	NICU	Blood	15	novel Ktype	R	I	R	R	R	R	R	R	R
10315_6#18	*Klebsiella pneumoniae*	28/6/2012	Nursery A	Blood	15	novel Ktype	R	I	R	R	R	R	R	R	R
10315_6#19	*Klebsiella pneumoniae*	26/6/2012	NICU	Blood	15	novel Ktype	R	R	R	R	R	R	R	R	R
10315_6#2	*Klebsiella pneumoniae*	17/5/2012	Medical	ET Tip	15	novel Ktype	R	R	R	R	R	R	R	R	R
10315_6#20	*Klebsiella pneumoniae*	29/6/2012	ICU	Sputum	15	novel Ktype	R	R	R	R	R	R	R	R	R
10315_6#21	*Klebsiella pneumoniae*	29/6/2012	Nursery A	Blood	15	novel Ktype	R	R	R	R	R	R	R	R	R
10315_6#22	*Klebsiella pneumoniae*	01/07/2012	ICU	Pus	340	K52	S	S	R	R	R	R	S	R	R
10315_6#23	*Klebsiella pneumoniae*	01/07/2012	ER	Swab	novel (47,1,13,3 ,12,25,18)	*R. Ornithininolytica* capsule	S	S	R	R	S	R	S	S	R
10315_6#24	*Klebsiella pneumoniae*	30/6/2012	ICU	Sputum	297	K10	S	S	R	S	S	S	S	S	R
10315_6#27	*Klebsiella pneumoniae*	06/07/2012	Medical	Catheter tip	307	NK29 (unidentified K type)	S	S	R	R	S	R	S	R	S
10315_6#28	*Klebsiella pneumoniae*	07/07/2012	Nursery C	Swab	15	K24	S	S	R	R	S	S	S	S	R
10315_6#29	*Klebsiella pneumoniae*	08/07/2012	PICU	ET Tip	15	*R. Planticola* capsule	S	S	R	R	R	S	S	S	R
10315_6#3	*Klebsiella pneumoniae*	24/5/2012	NICU	Blood	15	novel Ktype	R	R	R	R	R	R	R	R	R
10315_6#31	*Klebsiella pneumoniae*	11/07/2012	Medical	Sputum	147	K81	S	S	R	R	R	R	S	R	R
10315_6#32	*Klebsiella pneumoniae*	16/7/2012	Private	Urine	661	*K. oxytoca* E718	S	S	R	R	R	R	S	NR	R
10315_6#34	*Klebsiella pneumoniae*	19/7/2012	Medical	Sputum	661	*K. oxytoca* E718	S	S	R	R	S	R	S	NR	R

10315_6#38	*Klebsiella pneumoniae*	24/7/2012	Paediatric	Swab	1	K17	S	S	R	R	R	R	S	R	R
10315_6#39	*Klebsiella pneumoniae*	25/7/2012	Medical	Sputum	45	K24	S	S	R	I	S	S	S	S	S
10315_6#40	*Klebsiella pneumoniae*	25/7/2012	PICU	Sputum	716	untypeable	S	S	R	I	S	R	S	S	R
10315_6#41	*Klebsiella pneumoniae*	26/7/2012	Medical	Sputum	307	NK29 (unidentified K type)	S	S	R	R	R	R	S	S	R
10315_6#42	*Klebsiella pneumoniae*	29/7/2012	Medical	Sputum	661	K. oxytoca E718	S	S	R	R	S	R	S	R	R
10315_6#43	*Klebsiella pneumoniae*	31/7/2012	Gynaecological	Pus	14	untypeable	S	S	R	R	S	S	S	S	R
10315_6#44	*Klebsiella pneumoniae*	31/7/2012	Paediatric	Urine	novel (4,4,1,1,9, 1,4)	K30	S	S	I	S	S	S	S	NR	S
10315_6#45	*Klebsiella pneumoniae*	31/7/2012	Nursery B	Blood	14	K2	S	S	R	R	S	R	S	S	R
10315_6#49	*Klebsiella pneumoniae*	11/08/2012	Maternity	Pus	1559	K14	S	S	R	S	S	S	S	S	R
10315_6#5	*Klebsiella pneumoniae*	28/5/2012	NICU	ET Tip	15	novel Ktype	R	R	R	R	R	R	R	R	R
10315_6#51	*Klebsiella pneumoniae*	12/08/2012	Surgical	Tissue	307	NK29 (unidentified K type)	R	R	R	R	R	R	R	S	R
10315_6#52	*Klebsiella pneumoniae*	12/08/2012	Medical	Sputum	37	K12	S	S	R	I	S	S	S	S	R
10315_6#53	*Klebsiella pneumoniae*	16/8/2012	PICU	ET Tip	20	K28	S	S	R	I	S	S	S	S	R
10315_6#54	*Klebsiella pneumoniae*	16/8/2012	PICU	ET Tip	20	K28	S	S	R	I	S	S	S	S	NR
10315_6#57	*Klebsiella pneumoniae*	23/8/2012	Medical	Pus	15	novel Ktype	S	S	R	R	R	R	R	R	R
10315_6#58	*Klebsiella pneumoniae*	25/8/2012	Medical	Catheter tip	45	K52	S	S	R	S	S	R	S	R	R
10315_6#59	*Klebsiella pneumoniae*	26/8/2012	OPD	Urine	340	K52	S	S	R	R	R	S	S	NR	R
10315_6#6	*Klebsiella pneumoniae*	01/06/2012	NICU	Blood	15	novel Ktype	S	S	R	R	R	R	NR	R	R
10315_6#60	*Klebsiella pneumoniae*	26/8/2012	Medical	Urine	1	K17	S	S	R	R	R	R	S	NR	R
10315_6#61	*Klebsiella pneumoniae*	31/8/2012	Surgical	Tissue	35	K16	S	S	R	R	S	R	S	S	R
10315_6#62	*Klebsiella pneumoniae*	09/09/2012	ICU	Blood	15	K24	S	S	R	R	R	S	S	S	R
10315_6#65	*Klebsiella pneumoniae*	24/9/2012	Gynaecological	Urine	628	K52	S	S	R	R	S	R	S	NR	R
10315_6#66	*Klebsiella pneumoniae*	25/9/2012	Paediatric	Urine	15	novel Ktype	S	S	R	R	R	R	S	NR	R
10315_6#67	*Klebsiella pneumoniae*	26/9/2012	NICU	ET Tip	1559	K14	S	S	R	R	R	I	S	R	R
10315_6#68	*Klebsiella pneumoniae*	26/9/2012	NICU	Suction	1559	K14	S	S	R	R	R	S	S	R	R
10315_6#69	*Klebsiella pneumoniae*	27/9/2012	Maternity	Urine	711	K54	S	S	R	R	S	R	S	NR	R

10315_6#7	*Klebsiella pneumoniae*	03/06/2012	NICU	Blood	15	novel Ktype	S	S	R	R	R	R	R	R	R
10315_6#70	*Klebsiella pneumoniae*	28/9/2012	Private	Urine	novel (2,3,1,1,4, 4,4D)	untypeable	S	S	R	I	S	S	S	NR	R
10315_6#71	*Klebsiella pneumoniae*	29/9/2012	NICU	ET Tip	1559	K14	S	S	R	R	R	S	S	NR	R
10315_6#72	*Klebsiella pneumoniae*	29/9/2012	OPD	Pus	716	untypeable	S	S	R	I	S	R	S	S	S
10315_6#73	*Klebsiella pneumoniae*	29/9/2012	Postpartum	Urine	15	novel Ktype	S	S	R	R	R	R	S	S	R
10315_6#74	*Klebsiella pneumoniae*	30/9/2012	PICU	ET Tip	29	K19	S	S	R	R	S	S	S	S	R
10315_6#75	*Klebsiella pneumoniae*	01/10/2012	NICU	Blood	1559	K14	S	S	R	R	R	S	S	R	R
10315_6#76	*Klebsiella pneumoniae*	01/10/2012	Gynaecological	Urine	11	K52	R	R	R	R	R	R	R	NR	R
10315_6#77	*Klebsiella pneumoniae*	06/10/2012	PICU	ET Tip	15	novel Ktype	S	S	R	R	R	R	R	R	R
10315_6#78	*Klebsiella pneumoniae*	10/10/2012	Medical	Sputum	15	novel Ktype	S	S	R	R	R	R	S	S	R
10315_6#79	*Klebsiella pneumoniae*	10/10/2012	PICU	ET Tip	15	novel Ktype	S	S	R	R	R	R	R	R	R
10315_6#8	*Klebsiella pneumoniae*	09/06/2012	Nursery B	Blood	15	novel Ktype	R	I	R	R	R	R	R	R	R
10315_6#80	*Klebsiella pneumoniae*	11/10/2012	OPD	Pus	15	novel Ktype	R	I	R	R	R	R	R	R	R
10315_6#82	*Klebsiella pneumoniae*	14/10/2012	OPD	Urine	15	novel Ktype	S	S	R	R	R	R	R	NR	R
10315_6#85	*Klebsiella pneumoniae*	14/10/2012	ICU	Suction	551	K10	S	S	R	R	R	R	R	R	R
10315_6#87	*Klebsiella pneumoniae*	21/10/2012	Nursery B	Blood	1559	K14	S	S	R	R	R	S	S	R	R
10315_6#88	*Klebsiella pneumoniae*	28/10/2012	Maternity	Catheter tip	340	ST258-cps1	R	R	R	R	R	R	R	R	R
10315_6#89	*Klebsiella pneumoniae*	29/10/2012	PICU	Blood	1559	K14	S	S	R	S	S	S	S	S	S
10315_6#9	*Klebsiella pneumoniae*	13/6/2012	Nursery B	Blood	15	novel Ktype	R	I	R	R	R	R	R	R	R
10315_6#92	*Klebsiella pneumoniae*	05/11/2012	Nursery B	Blood	1559	K14	S	S	R	R	R	S	S	R	R
10315_6#93	*Klebsiella pneumoniae*	05/11/2012	Nursery B	Blood	1559	K14	S	S	R	R	R	S	S	R	R
10315_6#95	*Klebsiella pneumoniae*	07/11/2012	PICU	Blood	1559	K14	S	S	R	R	R	S	S	R	R
10315_6#96	*Klebsiella pneumoniae*	09/11/2012	Gynaecological	Urine	29	K19	S	S	R	R	S	S	S	NR	R
10356_5#76	*Klebsiella pneumoniae*	10/11/2012	Surgical	Urine	1559	K14	S	S	R	R	S	S	S	NR	R
10356_5#77	*Klebsiella pneumoniae*	12/11/2012	PICU	ET Tip	15	novel Ktype	S	S	R	R	R	R	R	R	R
10356_5#78	*Klebsiella pneumoniae*	12/11/2012	PICU	Blood	1559	K14	S	S	R	R	R	S	S	R	R
10356_5#79	*Klebsiella pneumoniae*	16/11/2012	ICU	Urine	152	untypeable	S	S	R	R	S	R	S	NR	R
10356_5#80	*Klebsiella pneumoniae*	18/11/2012	PICU	Blood	1559	K14	S	S	R	R	R	S	S	R	R
10356_5#81	*Klebsiella pneumoniae*	18/11/2012	PICU	Blood	441	K62	S	S	R	S	S	S	S	NR	R
10356_5#82	*Klebsiella pneumoniae*	18/11/2012	Nursery C	CSF	1559	K14	S	S	R	R	R	R	S	R	R

10356_5#85	*Klebsiella pneumoniae*	21/11/2012	PICU	Blood	1559	K14	S	S	R	R	R	I	I	R	R
10356_5#86	*Klebsiella pneumoniae*	22/11/2012	Maternity	Urine	25	K2	S	S	R	R	S	R	S	NR	R
10356_5#87	*Klebsiella pneumoniae*	26/11/2012	Maternity	Urine	4	K10	S	S	R	R	S	S	S	NR	R
9878_1#11	*Klebsiella pneumoniae*	21/11/2012	PICU	Blood	1559	K14	S	S	R	R	R	I	I	R	R
9878_1#12	*Klebsiella pneumoniae*	28/11/2012	Medical	Blood	1559	K14	S	S	R	R	R	R	S	R	R
9878_1#2	*Klebsiella pneumoniae*	01/06/2012	Nursery B	Blood	15	novel Ktype	R	R	R	R	R	R	R	R	R
9878_1#3	*Klebsiella pneumoniae*	08/06/2012	Nursery B	Blood	15	novel Ktype	R	I	R	R	R	R	R	R	R
9878_1#4	*Klebsiella pneumoniae*	28/6/2012	NICU	Blood	15	novel Ktype	R	R	R	R	R	R	R	R	R
9878_1#5	*Klebsiella pneumoniae*	30/6/2012	Nursery A	Blood	15	novel Ktype	R	I	R	R	R	R	R	R	R
9878_1#6	*Klebsiella pneumoniae*	03/07/2012	NICU	Blood	15	novel Ktype	R	R	R	R	R	R	R	R	R
9878_1#8	*Klebsiella pneumoniae*	30/9/2012	Nursery C	Blood	1559	K14	S	S	R	R	R	S	I	R	R
9878_1#9	*Klebsiella pneumoniae*	09/10/2012	Nursery C	Blood	1559	K14	S	S	R	R	R	S	NR	R	R

*Where the sequence type is novel, the allele profiles were attached in the following order (*gapA*, *infB*, *mdh*, *pgi, phoE*, *rpoB*, *tonB*), while 4D represents a divergent *tonB*4 allele (in 10315_6#70).

**Capsule genotyping was performed by comparing the *wzc* sequence to the Genbank database, known capsule type was recorded if the sequence identity was greater than 95%.

Antimicrobial resistance profile: MEM, meropenem; IMP, imipenem; CTX, ceftriaxone; CIP, ciprofloxacin; OFX, ofloxacin; GEN, gentamicin; AMK, amikacin; CHL, chloramphenicol; STX, trimethoprim/sulfamethoxazole; NR, not recorded.

Table S2. The primers used for retrospective PCR detection of Outbreak Lineages 1 and 2

Name	Reference	Primer sequence 5'-3'	Product (bp)	Target region
novK-F	This study	GGTCTGAAACGGGATATAGG	600	Specific beta-xylosidase within the K capsular region of Outbreak Lineage I
novK-R	This study	CGCACTATCACCACAAATG		
K14-F	This study	GACAACTGGCAAAGTCATAGG	400	Specific alginate lyase geme within the capsular region of K14 (ST1559 – Outbreak Lineage 2)
K14-R	This study	CCGAACAGGATTAAAGACTCC		
FIB-F	This study	GCAACAATGGACGTGTAGTT	1,000	Region spanning incF replication and upstream non-coding region, specific to pNDM-MAR
FIB-R	Villa et al., 2012	GTTACGATGGATGTGTCCC		
TnpA-F	This study	CGAATTGAGCGAAAAATTG	1,300	Region spanning TnpA transposon and NDM-1
NDM1-R	Poirel et al., 2011	CGGAATGGCTCATCACGATC		

Supplementary references

1 Villa L, Poirel L, Nordmann P, Carta C, Carattoli A. Complete sequencing of an IncH plasmid carrying the blaNDM-1, blaCTX-M-15 and qnrB1 genes. *J Antimicrob Chemother* 2012; **67**: 1645–50.

2 Poirel L, Walsh TR, Cuvillier V, Nordmann P. Multiplex PCR for detection of acquired carbapenemase genes. *Diagn Microbiol Infect Dis* 2011; **70**: 119–23.
