# Supplementary material for: A high-resolution genomic analysis of multidrug-resistant hospital outbreaks of Klebsiella pneumoniae
Source: EMBO Mol Med. 2015 Feb 20;7(3):227–39. doi: 10.15252/emmm.201404767 (PMC4364942; doi:10.15252/emmm.201404767)
Supplement: Supplementary file 2 [file emmm0007-0227-sd2.pdf]

EMBO Molecular Medicine

A high-resolution genomic analysis of multi-drug resistant hospital outbreaks of *Klebsiella pneumoniae*

Hao Chung The, Abhilasha Karkey, Duy Pham Thanh, Christine J Boinett, Amy K Cain, Matthew Ellington, Kate Baker, Sabina Dongol, Corinne Thompson, Simon R Harris, Thibaut Jombart, Tu Le Thi Phuong, Nhu Tran Do Hoang, Tuyen Ha Thanh, Shrijana Shretha, Suchita Joshi, Buddha Basnyat, Guy Thwaites, Nicholas R. Thomson, Maia A. Rabaa and Stephen Baker

Corresponding author: Stephen Baker, Hospital for Tropical Diseases, Wellcome Trust Major Overseas Programme, Oxford University Clinical Research Unit

Review timeline:

Submission date:	21 October 2014
Editorial Decision:	18 November 2014
Revision received:	23 December 2014
Editorial Decision:	16 January 2015
Accepted:	20 January 2015

Transaction Report:

(Note: With the exception of the correction of typographical or spelling errors that could be a source of ambiguity, letters and reports are not edited. The original formatting of letters and referee reports may not be reflected in this compilation.)

Editor: Céline Carret

1st Editorial Decision 18 November 2014

Thank you for the submission of your manuscript to EMBO Molecular Medicine. We have now heard back from the four referees whom we asked to evaluate your manuscript. Although they find the study to be of potential interest, they also raise significant issues that have to be addressed in the next version of your article.

As you will see from the reports pasted below, all referees find the study to be of potential interest, however the novelty aspect of the findings is compromised by existing literature not cited. While we still find the data suitable in principle for EMBO Molecular Medicine, this should be set right and the published findings and novelty aspect discussed appropriately. In addition, we would strongly encourage you to address all issues raised as recommended. As the referees' reports are very clear and nicely detailed I will not get into experimental details.

Please note that it is EMBO Molecular Medicine policy to allow only a single round of revision and that, as acceptance or rejection of the manuscript will depend on another round of review, your responses should be as complete as possible.

EMBO Molecular Medicine has a "scooping protection" policy, whereby similar findings that are published by others during review or revision are not a criterion for rejection. Should you decide to

submit a revised version, I do ask that you get in touch after three months if you have not completed it, to update us on the status.

Please also contact us as soon as possible if similar work is published elsewhere. If other work is published we may not be able to extend the revision period beyond three months.

Please see below for further information required upon revision submission.

I look forward to receiving your revised manuscript.

***** Reviewer's comments *****

Referee #1 (Remarks):

The study by The et al. reports the use of whole genome sequencing to characterize a hospital outbreak of Klebsiella pneumoniae in Kathmandu. In the end the authors found that the outbreak was cause by 2 separate lineages of K. pneumoniae. The work is well performed and contributes to our understanding of K. pneumoniae epidemiology and outbreaks, albeit the approach and findings are not especially novel. This is because previous studies, including one key study not mentioned here (Snitkin et al., Sci Transl Med., 2012), have used this approach to make similar observations with carbapenem-resistant K. pneumoniae. Specific comments are provided below.

1. The work by E.S. Sntikin et al. (Sci Transl Med, 2012) did essentially what was done in the current studies-used whole genome sequencing to retrospectively investigate the basis of a hospital outbreak of carbapenem-resistant K. pneumoniae. Resistance in the Snitkin study was conferred by KPC rather than NDM-1 as reported here, but the questions and approach were similar. Therefore, it is puzzling why the Snitkin study was omitted and not discussed/compared thoroughly by the authors. It was also highly publicized, so there are no excuses for this omission. Please revise the manuscript accordingly and tone down the conclusions as they relate to novelty. For example, the statement at the top of page 4 indicating "little is known about the genetic diversity...within a single hospital.", should be revised (along with all other such text).

2. Along those lines, another large-scale WGS study of carbapenem-resistant K. pneumoniae, (ref. Deleo et al.), identified a novel capsular polysaccharide synthesis (cps) region that contributed to lineage divergence. This seems similar to some of the findings here, but is given only a cursory-if not somewhat negative-mention. More notably, the authors of that study did do retrospective PCR analysis of the cps region, and used the approach as a tool for differentiation of isolates (Chen et al., AAC, 2014). Thus, the text on page 14 is inaccurate. More discussion of these and other relevant studies is needed, especially given the similarity of questions being asked and the similar general findings. I recommend that the authors perform a literature search and read the articles that are relevant to their work.

3. Please better define "core genome". The definition on page 5 does not indicate clearly whether mobile genetic elements were excluded. The subsequent text suggests they were omitted (at least in part), but clarification is needed.

Referee #2 (Remarks):

The authors describe a genomic analysis of an outbreak of Klebsiella pneumoniae (KP) infections that occurred in an hospital in Nepal in 2012.

By sequencing and subsequently analysing a large number of KP strains isolated in this hospital

over several months, before, during and after the outbreak, The spatio temporal resolution of the infection within the hospital could thus be revealed. Two major different and independent clone lineages responsible of the peak of blood infection were identified and characterised. The first one belongs to the ST15 and carries a new capsule operon. The second lineage belongs to ST1559 and presents a high degree of clonality. The authors then identified antimicrobial genes and putative virulence factors within these lineages and discuss their role in this outbreak.

The manuscript provides interesting detailed and new analysis on the spread of KP virulent strains in a hospital setting. However the manuscript can be improved and clarified in several ways, especially when talking to an audience non-necessarily specialist in microbial population genetics.

To my knowledge, virulent ST15 isolates are of K24 K-type. Interestingly, the authors observed that their ST15 isolates are of a new capsular serotype. However, the authors do not describe the presence of wzy, a polymerase gene conserved in capsule operon. Is it not clearly annotated or missing?

To which extend this new capsule serotype participates in the virulence is briefly discussed but not experimentally addressed. Comparing cell adhesion and resistance to serum and anti-microbial peptides of K24 ST15 isolate with the new ones would help understanding the role of this factor in the virulence. Similarly, how different are the 3 clones carrying the new capsular serotype mentioned in the discussion from the lineage 1 clones? Are they present in the clones shown in Figure 1? Do they fall on the same ST? carry similar virulence factors?

The ST and capsular type of the 39 strains that are not part of the 2 lineages (ST15 and ST1559) should be indicated in table S1.

Some characteristics of the ST15 are not clear specially when it comes to MDR plasmids and antimicrobials resistance :
page 6 indicates that 17/31 strains sequenced carry carbapenemase.
Figure 1C seems to show that 26/31 strains carried the plasmid pNDM-MAR, and 19/31 encodes for NDM1
While page 9 (line 232) it is said that "All of the Outbreak Lineage 1 organisms harboured a MDR plasmid, which shared a high degree of DNA homology to the previously described plasmid pNDM-MAR".
These numbers do not really match and are confusing. I don't get how they support the claim that the acquisition of carbapanemase is a main factor that drove the emergence of lineage 1 isolates.

The phylogenic analysis of the yersiniabactin described in Figure 4 does not seem to be relevant to the main message of the manuscript. I suggest to remove this part.

The PCR used to identify STs should be better described, and an example shown.

Has the 'comparative genomics and lineage specific gene content" resulting from the "accessory genome assembly' yield solely the K14 capsule operon as a difference from the reference strain 1084? This would be surprising. So why only the capsule operon was chosen to identify ST1559?

Similarly, can the authors use this sole criterium to identify the ST1559?

The description of the lineage specific gene content list the presence of knowns genes but does not highlight the new findings. As a distinctive T6SS is present in lineage 1 strains, the authors should describe and discuss the new features more clearly with a figure.

Other minor comments are below :

The authors mention (page 8) "several loci associated with attachment, including a biofilm encoding mrkABCD type 3 fimbriae locus" though only 1 is mentioned in table 1.

HGT acronym (page 8) is described only in page 9.

The description of the sequencing of non KP Gram negative bacteria falls aside the scope of this study and does not seem useful here.

I understand that the reads have been deposited to a database. Will an annotated version of the sequenced genomes (or the main ones) be available?

Some references are not fully described: eg, diancourt et al (2005) and Podschun and Ullmann (1998).

Referee #3 (Remarks):

The manuscript "A high resolution cross-sectional analysis of multi-drug resistant hospital outbreaks of Klebsiella pneumoniae" by Chung The et. al. compares clinical Klebsiella pneumoniae isolates collected during two independent outbreaks that occurred in high dependency wards of a hospital in Kathmandu in 2012. The authors use whole genome sequencing (W.G.S.) in addition to antibiotic resistance testing, classical PCR and metadata to demonstrate that these two outbreaks were the result of independent isolate lineages and argue that the implementation of W.G.S. for routine isolate typing would enable clinicians to investigate outbreaks and take the appropriate measures in a timely manner.

Major Comments

While the scientific background and results described in this manuscript are worth publication and would benefit the scientific community, the authors should review the discussion and the focus of their paper. W.G.S. is indeed a powerful technique for epidemiological typing of outbreak isolates but its implementation as a routine technique is currently not feasible, especially in low-income countries as described here and pointed out by the authors themselves. In particular, a discussion about the software requirements, cost, server capabilities, processing and analysis time would be useful.

What are the advantages of W.G.S. over typing methods such as MLST and PFGE for infection control and outbreak investigations?

How does knowing that the strains acquired yersiniabactin and pNDM-MAR effect infection control?

Minor Comments

1. Line 65: The word "as" is missing.
2. Line 71: "mobile carbapenemase" should be replaced by "mobile genetic elements carrying carbapenemase resistance genes"
3. Figure 1 should be simplified and reduced to include only what the authors discuss in the results section. The authors might also want to comment on the temporal characteristic of these outbreaks. Data on a longer time period might be interesting if these cycles are recurrent. In particular, line 107 refers to 2011 data which should also be shown in Figure 1b.
4. Figure 1b and c: Please align labels (months and antibiotics).
5. Figure 1 legend: Please define "MON".
6. Line 146: "data not shown" - It may help the readers to include this information.
7. Lines 194-223: Throughout most of the manuscript, the authors discuss Lineage 1 and then Lineage 2. However, in this section, the authors describe Lineage 2 and then Lineage 1. For consistency and to make it easier for the reader, it would be helpful to swap these two paragraphs (I.e., describe Lineage 1 and then Lineage 2).
8. Lines 194-223 and Table 1: Are all of the genes identified unique to Lineage 1 or 2? Please clarify.
9. Figure 2 legend: Please define "OPD".

10. Figure 2a: The authors might want to comment on the fact that adult K. pneumoniae isolates segregate from children's isolates, which might be related to host specificity and not geographical distance.
11. Figure 2b does not add anything and could be removed.
12. Line 206: Please define "HGT".
13. Line 277-278: What treatment did the patients receive during the first outbreak?
14. Lines 312-33: Do the authors have any phenotypic data to support this statement?
15. Line 314: Do the authors mean iron "chelation"?
16. Lines 343 to 360: the authors should rewrite this paragraph. Alternative techniques that are simpler, faster and more economical exist for epidemiological tying of clinical isolates. For example, the data indicates that MLST would have been sufficient to differentiate the outbreak strains. What are the advantages of W.G.S.?
17. Table 1: It would be useful to list the gene locations/ORF label for each genetic element.

Referee #4 (Remarks):

The authors describe the genomic dissection of two consecutive outbreaks of invasive (bloodstream) infections due to Klebsiella pneumoniae in one Nepalese hospital. The outbreaks involved many patients and had a very high fatality rate, underlining the importance of investigating the causative strains to better understand transmission, evolution and pathogenesis.
The authors deployed whole genome sequencing, phylogenetic methods, genomic content comparisons on a randomly selected sample of the outbreaks period and went further to develop PCR tests derived from unique genomic regions to investigate large numbers of strains previous to the outbreak period to test anterior presence of the outbreak lineages.

This is a large, in-depth, well-presented, high-quality analysis in terms of sequencing, phylogenetics and PCR characterization of hospital strains. Arguably the first study of its kind in low-resources countries (but the authors omitted to mention the Snitkin et al study of a 2011 hospital outbreak of MDR K. pneumoniae in the USA). Two lineages were associated with the two outbreak peaks. One unexpected and interesting finding is that sublineages of the outbreak strains have been restricted to particular wards, a conclusion that was possible only due to the high resolution provided by WGS. One minor weakness is the root-to-tip regression analysis due to severe lack of informative nucleotide variation and time span, but this is appropriately discussed by the authors, and it is remarkable that the authors defined unique PCR regions and screened with for the presence of these regions over 400 additional strains to define how long the outbreak lineages were present in the hospital. The removal of recombination regions is also highly appropriate for phylogenetic analysis of these closely related strains, but here it would be good if the authors would provide some details of the method. The figures are of high information content and quality. Overall it was a pleasure to read the manuscript, which is highly informative, both in methodology and content.

The authors also characterized the resistance gene complement of the strains from their genomes in an appropriate way. I wondered how they defined plasmid versus chromosomal borne elements as their assemblies are fragmented, and the decision rules should be stated more explicitly (homology to known plasmids I would guess?).

I would be slightly more critical on the comparison of pathogenicity elements, as this part has several weaknesses. First, comparative strains used to define unique pathogenicity elements or genomic features were not presented, unless I missed them; so it is very hard to understand how unique the elements of lineages 1 and 2 are. Was a reference set of publicly available genomes used? Second, it is unclear which list of virulence factors was screened and why some features were described and others not. For example, the authors chose to pick the mrk cluster coding for type III fimbriae but not the fim cluster also known to be involved in pathogenesis, while both are highly conserved among K. pneumoniae strains. Same for enterobactin. I think the paper would benefit from more clarity in these aspects.

Some reorganization of the results section seems to be needed. The 'Assessing the temporal scale' paragraph could first describe the diversity/root-to-tip approach both both lineages ; then the PCR approach for both lineages. The 'comparative genomics ...' section starts with lineage 2, why not by lineage 1 as previously? It includes some data on MDR genes and resistance phenotypes and this could more appropriately be moved to the next 'resistance' section. And again the list of comparative genomes used in the comparative genomics section should be stated (for example lines 194-195). Or is it not 'comparative' but just mining the lineage 1 and 2 genomes?

Specific points

Title: is 'cross-sectional' clear enough to appear in the title? I personally am not sure what this means.

Line 56: urinary tract infections are the most common in many settings.

Lines84-86: this sentence seems to suggest that not much is currently available to type Kp strains; MLST and PFGE could be mentioned in the introduction ; even though I agree that in many settings only very crude methods are in use.

Lines 105: PICU and NICU might be defined here?

Lines 126: A few words on the sequencing method, strain set and assembly should be provided here before describing the core genome.

Line 129: it would be informative to state the max and min % nucleotide distance among strains; were all strains clearly belonging to a single species?

Line 139: but Bialek et al. EID 2014 recently showed ST14 and ST15 belong to distinct clonal groups.

Line 140: is ST1559 phylogenetically closely related to known carbapenem-producing clones such as ST258 or ST11?

Lines 171 - 174: these molecular markers of lineage 1 are questionable as they are mobile elements or prone to HGT. Implications should be commented/could other strains harboring these markers have been misidentified as lineage 1 by this PCR strategy?

Line 172: Novel capsular type and Figure 3: surprisingly there is no wzy polymerase gene identified. Probably the hypothetical protein before wzx (why was this gene call wzxC)? Authors could hunt for conserved motives of wzy or use relaxed BLAST criteria, as this is a highly variable gene but always present in cps clusters.

Line 198: 'were of capsule type 14': as deduced based on the genomic sequence? Based on which criteria? Or was antiserum used to confirm the serotype?

Line 199: 'similar' is a bit vague; move this to next section.

Line 204-205: yersiniabactin is not present in all Kp strains according to literature. What is meant with 'common': frequent?

I found the yersinibactin phylogenetic analysis not really useful here and a bit out of scope; unless to say that both lineages acquired it independently, which is useful. Also it was unclear to me how the phylogeny was constructed: concatenated alignments of all CDSs of the cluster? If so, were they congruent among themselves or is there some kind of mosaicism?

Line 212: what is meant with 'contemporary'? Seems strange to me here.

Line 213: 'orientation': organization meant? What about other cps clusters: are they not typically of low CG%?

Line 217 'from Kp 342': supported by very high degree of similarity?

Line 220: not sure that kfu is a siderophore system (and also fec)

Line 232: 'resistance plasmid': how many contigs/ fully covered? How do you know it is a single plasmid?
Line 232: 'high degree': how much precisely?
Line 235: 'excision' more likely possibly, but can it instead be gain of the NDM1 in the other strains?

Line 244 'impacting polymixin susceptibility': possibly contradicts line 231/ what about colistin?

Line 260: it should be fair to quote Snitkin et al here.

Lines 291-300: not totally clear to me...

Line 312: 'these siderophores': which ones? Could quote here, Bachman work demonstrating how yersiniabactin is useful in complement to other siderophores.

Line 321 'cross sectional': what is meant (again!)?

Line 322: how was CC14 determined if ST was not provided? CC definition is difficult in kp due to lack of clear discontinuities - so this is possibly a weak argument to say strains are closely related.

Line 347: Ramos et al was not an outbreak investigation; Snitkin et al is missing here.

Line 350 - 360: the authors turn a bit lyric about WGS here - I would tend to think that other typing methods could have been used e.g. a simple ERIC-PCR screen, MLST, PFGE, would all have identified/distinguished the 2 outbreak strains. I fully agree that WGS was key to dissect the outbreaks though. Maybe the authors would want to distinguish strain typing /surveillance on the one hand, and in-depth phylogenetic and genomic analyses rendered possible by WGS on the other hand.

Lines 419-420: no processing of the raw reads (quality...) was performed before assembly?

Lines 445-455: not sure I understood the process. What are the advantages of the strategy used here compared to classical protein cluster construction for accessory genome definition?

Line 466 'constant size demographic model': this seems inconsistent with the fact that outbreak strains were included, as a population expansion would have been expected. No?

Line 481: 'rule' without 'd' I guess. Were all genes of the HPI cluster considered together?

Lines 492-494: please provide polymerase/ reagent/ buffer/ kit used.

Figure 1 legend: 'MON' should be defined. Black arrow: only one lineage so no 's'. 'with a two mutations': ? Scale bar seems to imply many nucleotide differences among lineages. Is it really correct?
Would be good to provide ST of all branches in Figure 1; possibly in Table S1? Are the branches closely related to ST15 and ST1559 (each have a small sister group) also ST15 and ST1559?

Figure 2 legend: OPD meaning? Why are there black dots right of the last black arrow?

Figure 3 legend: 'orientation': of the genes meant?

Figure 4 legend: only one bootstrap value shown it seems but plural used in legend?

Figure 5: please name the genes in the legend. What is mph?

Data Accessibility: were sequences submitted to public databases (eg novel capsule cluster)?

1st Revision - authors' response 23 December 2014

Referee #1 (Remarks):

The study by The et al. reports the use of whole genome sequencing to characterize a hospital outbreak of Klebsiella pneumoniae in Kathmandu. In the end the authors found that the outbreak was cause by 2 separate lineages of K. pneumoniae. The work is well performed and contributes to our understanding of K. pneumoniae epidemiology and outbreaks, albeit the approach and findings are not especially novel. This is because previous studies, including one key study not mentioned here (Snitkin et al., Sci Transl Med., 2012), have used this approach to make similar observations with carbapenem-resistant K. pneumoniae. Specific comments are provided below.

We thank the reviewer for the encouraging comments and acknowledge the oversight of the Snitkin paper and have now added and discussed the reference appropriately in the manuscript.

The work by E.S. Sntikin et al. (Sci Transl Med, 2012) did essentially what was done in the current studies-used whole genome sequencing to retrospectively investigate the basis of a hospital outbreak of carbapenem-resistant K. pneumoniae. Resistance in the Snitkin study was conferred by KPC rather than NDM-1 as reported here, but the questions and approach were similar. Therefore, it is puzzling why the Snitkin study was omitted and not discussed/compared thoroughly by the authors. It was also highly publicized, so there are no excuses for this omission. Please revise the manuscript accordingly and tone down the conclusions as they relate to novelty. For example, the statement at the top of page 4 indicating "little is known about the genetic diversity...within a single hospital.", should be revised (along with all other such text).

See above comment. We agree that whole genome sequencing was applied to study a K. pneumoniae outbreak in the previous studies (Snitkin et al., 2012). However, they did not investigate the genetic diversity of the K. pneumoniae population while taking into account non-outbreak strains isolated in the same hospital setting.

Snitkin et al. used whole genome sequencing on 18 outbreak strains to construct a most likely transmission chain. The novelty of our study is that we include multiple strains of a multitude of sample types (include non-outbreak strains) to study the genetic diversity as well as the antibiotic resistance profile of the population. This also permits us to use comparative genomics to study the genomic discrepancy of the outbreak in its population context. We have added the reference and edited the passage to read "The genetic diversity of K. pneumoniae causing hospital outbreaks has been studied previously, with the primary focus being particular emergent clones in affluent settings (Brisse et al, 2009; Snitkin et al, 2012; Deleo et al, 2014). There is currently limited knowledge regarding the overall genetic diversity of this major pathogen within a single hospital setting (including non-outbreak isolates). This limitation is highly pertinent in resource-poor settings where these infections may exhibit more antimicrobial resistance and cause particularly aggressive infections." (page 4, lines 80-85).

Along those lines, another large-scale WGS study of carbapenem-resistant K. pneumoniae, (ref. Deleo et al.), identified a novel capsular polysaccharide synthesis (cps) region that contributed to lineage divergence. This seems similar to some of the findings here, but is given only a cursory-if not somewhat negative-mention. More notably, the authors of that study did do retrospective PCR analysis of the cps region, and used the approach as a tool for differentiation of isolates (Chen et al., AAC, 2014). Thus, the text on page 14 is inaccurate. More discussion of these and other relevant studies is needed, especially given the similarity of questions being asked and the similar general findings. I recommend that the authors perform a literature search and read the articles that are relevant to their work.

Deleo et al. is another outstanding study in K. pneumoniae genomics, but its context is neither an outbreak investigation nor inclusive of background hospital strains. We have discussed this alongside with Chen et al., 2014 study in the discussion. We acknowledge that these studies focus on the KPC-producing ST258, and our study complements this by improving understanding of NDM-1 producing ST15 clones. This has been addressed (page 15, lines 384-388).

Please better define "core genome". The definition on page 5 does not indicate clearly whether mobile genetic elements were excluded. The subsequent text suggests they were omitted (at least in part), but clarification is needed.

The core genome was defined as the set of protein coding sequences found in a single copy in all strains. There are two sequenced strains that had no sequence based evidence of plasmids (based on comparison with reference genome), so the core genome contains minimal mobile genetic elements. Further, we removed any putative regions of recombination in the alignment based on the clustering of SNP density (as mentioned in the Methods). This has been edited in the Results section (page 6, lines 142-149).

Referee #2 (Remarks):

Corresponding author's responses are italicized and indented

The authors describe a genomic analysis of an outbreak of Klebsiella pneumoniae (KP) infections that occurred in a hospital in Nepal in 2012. By sequencing and subsequently analysing a large number of KP strains isolated in this hospital over several months, before, during and after the outbreak, The spatio temporal resolution of the infection within the hospital could thus be revealed. Two major different and independent clone lineages responsible of the peak of blood infection were identified and characterised. The first one belongs to the ST15 and carries a new capsule operon. The second lineage belongs to ST1559 and presents a high degree of clonality. The authors then identified antimicrobial genes and putative virulence factors within these lineages and discuss their role in this outbreak.

The manuscript provides interesting detailed and new analysis on the spread of KP virulent strains in a hospital setting. However the manuscript can be improved and clarified in several ways, especially when talking to an audience non-necessarily specialist in microbial population genetics.

We thank the reviewer for their insightful comments

To my knowledge, virulent ST15 isolates are of K24 K-type. Interestingly, the authors observed that their ST15 isolates are of a new capsular serotype. However, the authors do not describe the presence of wzy, a polymerase gene conserved in capsule operon. Is it not clearly annotated or missing?

This was an oversight. The "O-antigen ligase" gene is indeed wzy, and this was confirmed by Pfam search to show the presence of the Wzy_C domain in the protein. This has now been annotated in Figure 3. Out of 31 ST15 strains in our study, 28 belong to this novel capsular

type while only 2 are of K24 (Table S1). We have compared this novel whole capsule region as well as gene wzc to sequences in the Genbank database and confirmed its novelty. ST15 is a global clone with great diversity, so the acquisition of a new capsular type is not entirely surprising.

To which extend this new capsule serotype participates in the virulence is briefly discussed but not experimentally addressed. Comparing cell adhesion and resistance to serum and anti-microbial peptides of K24 ST15 isolate with the new ones would help understanding the role of this factor in the virulence. Similarly, how different are the 3 clones carrying the new capsular serotype mentioned in the discussion from the lineage 1 clones? Are they present in the clones shown in Figure 1? Do they fall on the same ST? carry similar virulence factors?

We agree that this would be interesting but we feel it is outside of the scope of this current clinical/epidemiological analysis. The 3 strains carrying the new capsular type mentioned in the discussion belong to ST15, which are shown in Figure 1. They are similar to Outbreak Lineage 1 in terms of gene content except for the absence of pNDM-MAR plasmid, pCTX-M3 integron, pRAS2 strAB, and ICE-KpNp1. This has been addressed in the text (page 13, lines 332) and in Table 1.

The ST and capsular type of the 39 strains that are not part of the 2 lineages (ST15 and ST1559) should be indicated in table S1.

This has now been added to Table S1.

Some characteristics of the ST15 are not clear specially when it comes to MDR plasmids and antimicrobials resistance:

Page 6 indicates that 17/31 strains sequenced carry carbapenemase.

Figure 1C seems to show that 26/31 strains carried the plasmid pNDM-MAR, and 19/31 encodes for NDM1

While page 9 (line 232) it is said that "All of the Outbreak Lineage 1 organisms harboured a MDR plasmid, which shared a high degree of DNA homology to the previously described plasmid pNDM-MAR".

These numbers do not really match and are confusing. I don't get how they support the claim that the acquisition of carbapanemase is a main factor that drove the emergence of lineage 1 isolates.

We agree, this is confusing and has been edited throughout to clarify. There were 25 Outbreak Lineage 1 isolates – this is defined by the novel capsule cluster and pNDM-MAR plasmid. 19/25 contained NDM-1.

The phylogenic analysis of the yersiniabactin described in Figure 4 does not seem to be relevant to the main message of the manuscript. I suggest removing this part.

We have removed this portion of the analysis for the sake of clarity. Figure 4 has also been removed at the reviewer's request.

The PCR used to identify STs should be better described, and an example shown.

Has the 'comparative genomics and lineage specific gene content" resulting from the "accessory genome assembly' yield solely the K14 capsule operon as a difference from the reference strain

1084? This would be surprising. So why only the capsule operon was chosen to identify ST1559? Similarly, can the authors use this sole criterium to identify the ST1559?

We have included more details of the PCR targets in the text and the primers are shown in Table S2. A genomic comparison of the ST1559 (Outbreak Lineage 2 isolates) with the remaining strains in our study revealed several unique regions, including the capsule region, several prophages, etc. However, we selected the specific capsule type K14 because of its clinical importance, and previous studies have characterized the clonality of K. pneumoniae based on PCR of the capsule region. For our study we inferred the ST1559 strains by PCR amplification of a capsule gene. We agree that extensive transfer of this capsule could compromise this inference – yet the results were consistent with the extent of this second outbreak. However, this has been mentioned as a limitation in our discussion (page 15, lines 376-382).

The description of the lineage specific gene content list the presence of known genes but does not highlight the new findings. As a distinctive T6SS is present in lineage 1 strains, the authors should describe and discuss the new features more clearly with a figure.

The T6SS described in Table 1 is not exclusive to Outbreak Lineage 1 (Table 1). We apologize for this confusion. We have now highlighted the elements that are novel with respect to Outbreak Lineage 1 and 2 in Table 1. We have been asked to remove the Yersiniabactin and other comparative genomics by yourself and other reviewers. Highlighting other genomic elements with a figure may not improve the clarity of what we are trying to present.

Other minor comments are below:

The authors mention (page 8) "Several loci associated with attachment, including a biofilm encoding mrkABCD type 3 fimbriae locus" though only 1 is mentioned in table 1.

There is more that one element associated with attachment in Table 1. We have edited the text to read 'multiple' rather than 'several' (page 10, line 252).

HGT acronym (page 8) is described only in page 9.

This has been edited (page 12, line 308).

The description of the sequencing of non KP Gram negative bacteria falls aside the scope of this study and does not seem useful here.

Information pertaining to these strains has now been removed.

I understand that the reads have been deposited to a database. Will an annotated version of the sequenced genomes (or the main ones) be available?

Yes all of the read information has been deposited into the European Nucleotide Archive (ENA) under the accession numbers ERR349747 to ERR349855. The assemblies of the two representatives of the two outbreaks have been registered as PRJEB8009 (lineage 1) and PRJEB8010 (lineage 2), and are awaiting final deposition. The capsule biosynthesis cluster

has been submitted and has accession number LN714331.

Some references are not fully described: eg, diancourt et al (2005) and Podschun and Ullmann (1998).

These have been edited accordingly

Referee #3 (Remarks):

The manuscript "A high resolution cross-sectional analysis of multi-drug resistant hospital outbreaks of Klebsiella pneumoniae" by Chung The et. al. compares clinical Klebsiella pneumoniae isolates collected during two independent outbreaks that occurred in high dependency wards of a hospital in Kathmandu in 2012. The authors use whole genome sequencing (W.G.S.) in addition to antibiotic resistance testing, classical PCR and metadata to demonstrate that these two outbreaks were the result of independent isolate lineages and argue that the implementation of W.G.S. for routine isolate typing would enable clinicians to investigate outbreaks and take the appropriate measures in a timely manner.

Major Comments

While the scientific background and results described in this manuscript are worth publication and would benefit the scientific community, the authors should review the discussion and the focus of their paper. W.G.S. is indeed a powerful technique for epidemiological typing of outbreak isolates but its implementation as a routine technique is currently not feasible, especially in low-income countries as described here and pointed out by the authors themselves. In particular, a discussion about the software requirements, cost, server capabilities, processing and analysis time would be useful. What are the advantages of W.G.S. over typing methods such as MLST and PFGE for infection control and outbreak investigations?

We thank the reviewer for their valuable comments.

It is difficult to get the message correct here. In fact the major issue is actually buying and running the machine. PFGE gives poor data given the effort required and MLST profiling is actually more expensive than WGS now, when taking the cost of amplification and conventional sequencing of the seven loci in both directions in account. Indeed we are now running a MiSeq to address these problems.

An additional reviewer raised similar concerns and we have toned the message regarding WGS and suggested that some form of routine genetic characterization would have added some information that could have been interpreted for the infection control. We think that this is a little less controversial (page 16, lines 399-411).

How does knowing that the strains acquired yersiniabactin and pNDM-MAR effect infection control?

We have toned down the discussion regarding specific genetic markers and their use for identifying virulent clones. Additionally, we have been asked to remove the majority of the

yersiniabactin data.

The point we were trying to make was that in this setting bacteremia is relatively common in these wards. The wards are in a poor condition and the children infected are in a high-risk group. Better infection control would improve this. However, having multiple bacteremia cases caused by an NDM-1 containing K. pneumonia of the same lineage should point to the fact that this is an outbreak and that it is being transmitted from patient-to-patient. We perhaps were thinking from a bacteriology standpoint and the effect of virulence factors and resistance gene on disease phenotype and treatment. Detecting strains early with an increase range of AMR genes and novel virulence genes (compared to background strains) would be beneficial in understanding why the outbreak strains had an aggressive phenotype.

We hope that the edited discussion is more in line with your thoughts on this subject.

Minor Comments

Line 65: The word "as" is missing.

This has now been added (page 3, line 65).

Line 71: "mobile carbapenemase" should be replaced by "mobile genetic elements carrying carbapenemase resistance genes"

This has now been edited (page 3, line 72).

Figure 1 should be simplified and reduced to include only what the authors discuss in the results section. The authors might also want to comment on the temporal characteristic of these outbreaks. Data on a longer time period might be interesting if these cycles are recurrent. In particular, line 107 refers to 2011 data, which should also be shown in Figure 1b.

We have edited the text to address this issue. The comment regarding 2011 has been removed. The figure now corresponds precisely with the presented results (page 5, line 112). We do not have good surveillance data outside 2012 to address cyclical trends.

Figure 1b and c: Please align labels (months and antibiotics).

This has been edited this accordingly.

Figure 1 legend: Please define "MON".

This is now defined as "month of isolation".

Line 146: "data not shown" - It may help the readers to include this information.

We have three phylogenetic trees in the manuscript already and the authors think that a

fourth would not add anything to the scope of the manuscript; all this information is summarized in Figure 2.

Lines 194-223: Throughout most of the manuscript, the authors discuss Lineage 1 and then Lineage 2. However, in this section, the authors describe Lineage 2 and then Lineage 1. For consistency and to make it easier for the reader, it would be helpful to swap these two paragraphs (I.e., describe Lineage 1 and then Lineage 2).

We have rearranged the text accordingly (pages 9-11).

Lines 194-223 and Table 1: Are all of the genes identified unique to Lineage 1 or 2? Please clarify.

Not all genes in Table 1 are unique to Lineage 1 or 2. The presence of these genes is shown in Table 1. We have additionally edited the Methods and Results for clarity (page 9, 229-233; page 20-21, line 519-526)

Figure 2 legend: Please define "OPD".

This has now been defined as the outpatient department.

Figure 2a: The authors might want to comment on the fact that adult K. pneumoniae isolates segregate from children's isolates, which might be related to host specificity and not geographical distance.

Although this is an interesting concept we feel that this is beyond the scope of the findings of this study. Our samples were not collected to test this very specific hypothesis and would likely lack sufficient power to answer that with any degree of certainty. Therefore we would not like to speculate on this in the manuscript.

Figure 2b does not add anything and could be removed.

We included Figure 2b to show that the temporal signal in our sequences was limited and wish to leave for clarity; this has been addressed by reviewer 4 and would be questioned by phylogeneticists if removed. If the manuscript is accepted we are willing to remove this at the editor's request.

Line 206: Please define "HGT".

This has been now been defined (page 12, line 308).

Line 277-278: What treatment did the patients receive during the first outbreak?

During the first outbreak the children initially received meropenem and were latterly changed onto colistin. However, hospital records are limited and we are unable to specifically define treatment regimes and durations. This is a limitation, but highlights the problems of routine data capture in such a setting. We think that referring to treatment

without the precise data would be non-scientific and unhelpful.

Lines 312-33: Do the authors have any phenotypic data to support this statement?

No, we do not have any laboratory data to support and it is a hypothesis given the data. We have rewritten this section for clarification "We speculate that the excess of these iron acquisition systems contributed greatly to the ability of both outbreak lineages of K. pneumoniae to cause systemic infections, potentially by allowing more efficient replication whilst in the bloodstream through enhanced iron chelation" (page 14, lines 348-351).

Line 314: Do the authors mean iron "chelation"?

Indeed – this has been edited (page 14, line 351).

Lines 343 to 360: the authors should rewrite this paragraph. Alternative techniques that are simpler, faster and more economical exist for epidemiological tying of clinical isolates. For example, the data indicates that MLST would have been sufficient to differentiate the outbreak strains. What are the advantages of W.G.S.?

As above – this section has now been re-written to address these concerns. We have tried to make a differentiation between WGS and routine surveillance. Another reviewer raised this question.

Table 1: It would be useful to list the gene locations/ORF label for each genetic element.

This cannot be done as the sequence data was generated using illumina technology and then aligned to a reference sequence; therefore we cannot infer the exact locations of these elements.

Referee #4 (Remarks):

The authors describe the genomic dissection of two consecutive outbreaks of invasive (bloodstream) infections due to Klebsiella pneumoniae in one Nepalese hospital. The outbreaks involved many patients and had a very high fatality rate, underlining the importance of investigating the causative strains to better understand transmission, evolution and pathogenesis.

The authors deployed whole genome sequencing, phylogenetic methods, genomic content comparisons on a randomly selected sample of the outbreaks period and went further to develop PCR tests derived from unique genomic regions to investigate large numbers of strains previous to the outbreak period to test anterior presence of the outbreak lineages.

This is a large, in-depth, well-presented, high-quality analysis in terms of sequencing, phylogenetics and PCR characterization of hospital strains. Arguably the first study of its kind in low-resources countries (but the authors omitted to mention the Snitkin et al study of a 2011 hospital outbreak of MDR K. pneumoniae in the USA).

We thank the reviewer for their encouragement and recognize the error in omitting the Snitkin study. This has now been addressed. We now discuss this reference in both the introduction and the discussion (page 4, line 80-82; page 12, line 297).

Two lineages were associated with the two outbreak peaks. One unexpected and interesting finding is that sublineages of the outbreak strains have been restricted to particular wards, a conclusion that was possible only due to the high resolution provided by WGS. One minor weakness is the root-to-tip regression analysis due to severe lack of informative nucleotide variation and time span, but this is appropriately discussed by the authors, and it is remarkable that the authors defined unique PCR regions and screened with for the presence of these regions over 400 additional strains to define how long the outbreak lineages were present in the hospital.

Yes, we recognized this limitation and addressed it experimentally in the most appropriate manner possible, we felt it would be better to fully disclose the data and its limitations rather than use BEAST inappropriately and misinterpret the results as in other bacterial studies. Many thanks for recognizing this! – it was a dilemma before submission.

The removal of recombination regions is also highly appropriate for phylogenetic analysis of these closely related strains, but here it would be good if the authors would provide some details of the method. The figures are of high information content and quality. Overall it was a pleasure to read the manuscript, which is highly informative, both in methodology and content.

Again. Many thanks for the support.

We have provided some details of the method to remove recombination (page 19, lines 490-494). For more details, please refer to Croucher et al., 2014.

The authors also characterized the resistance gene complement of the strains from their genomes in an appropriate way. I wondered how they defined plasmid versus chromosomal borne elements as their assemblies are fragmented, and the decision rules should be stated more explicitly (homology to known plasmids I would guess?).

Genetic elements were queried to the database using BLAST. Elements were classified as located in a plasmid if they showed homology (identity >= 95%) to existing plasmids in the database. Elements were classified as chromosome-borne if they showed homology to known chromosomal regions. This information has now been added to the Methods. (page 20-21, lines 519-526).

I would be slightly more critical on the comparison of pathogenicity elements, as this part has several weaknesses. First, comparative strains used to define unique pathogenicity elements or genomic features were not presented, unless I missed them; so it is very hard to understand how unique the elements of lineages 1 and 2 are. Was a reference set of publicly available genomes used? Second, it is unclear which list of virulence factors was screened and why some features were described and others not. For example, the authors chose to pick the mrk cluster coding for type III fimbriae but not the fim cluster also known to be involved in pathogenesis, while both are highly conserved among K. pneumoniae strains. Same for enterobactin. I think the paper would benefit from more clarity in these aspects.

The presence of elements in Table 1 has been added, and not all are unique to either lineage.

We apologize for this confusion.

The comparative genomics utilized in this study were designed to identify unique regions present in each outbreak lineage compared to the non-outbreak background strains. This helps us to explain the virulence of these strains in the context of this study. Therefore, we did not use other published reference genomes for exhaustive comparison (except for K. pneumoniae 1084 as included in the Methods section). Not all features detected from comparative genomics were indicative of virulence (for example, some unique regions are uncharacterized phage-borne). We only selected genetic elements that are potential virulence factors based on functionality (fimbriae, capsule type, siderophores, etc.) to explain the virulence of these outbreak strains. We also searched all 89 K. pneumoniae genomes determined in this study for known virulence factors using BLAST, and we did not consider factors that are prevalent in all or almost all strains. These include enterobactin (found in all), fim1 cluster (found in all). This ensures that candidates in Table 1 represent both uniqueness (to some degree) and potential virulence. This has been added to the Methods and Results sections for clarity (page 20-21, line 519-526; page 9, 229-233).

Some reorganization of the results section seems to be needed.

The 'Assessing the temporal scale' paragraph could first describe the diversity/root-to-tip approach both lineages ; then the PCR approach for both lineages.

This has been edited to reflect this comment (pages 8-9).

The 'comparative genomics ...' section starts with lineage 2, why not by lineage 1 as previously? It includes some data on MDR genes and resistance phenotypes and this could more appropriately be moved to the next 'resistance' section.

We have reorganized this section so that it better follows the structure of the rest of the paper, describing Lineage 1 followed by Lineage 2. We have deliberately focused on lineage 1 as is more interesting than lineage 2. We have edited the section titles to clarify (pages 9-11).

And again the list of comparative genomes used in the comparative genomics section should be stated (for example lines 194-195). Or is it not 'comparative' but just mining the lineage 1 and 2 genomes?

This has been edited "Aiming to understand the genetic basis for the phenotypes associated with Outbreak Lineage 1 and Lineage 2, we performed comparative genome analyses by aligning the gene content of the two Outbreak Lineages strains against the background hospital strains" (page 9, 229-233).

Specific points

Title: is 'cross-sectional' clear enough to appear in the title? I personally am not sure what this means.

The term cross-sectional has been omitted from the title.

Line 56: urinary tract infections are the most common in many settings.

This has been added (page 3, line 56).

Lines84-86: this sentence seems to suggest that not much is currently available to type Kp strains; MLST and PFGE could be mentioned in the introduction; even though I agree that in many settings only very crude methods are in use.

This has been edited "This knowledge gap is due in part to a lack of sensitive phenotypic and molecular tools for accurately differentiating or subtyping K. pneumoniae. For most Gram-negative bacteria, relatedness among isolates has traditionally been deduced using antimicrobial susceptibility patterns, crude microbiological methods or molecular typing methods such as Pulsed Field Gel Electrophoresis (PFGE) or Multi Locus Sequence Typing (MLST). (page 4, lines 85-89)

Lines 105: PICU and NICU might be defined here?

These have now been defined (page 5, line 110-111).

Lines 126: A few words on the sequencing method, strain set and assembly should be provided here before describing the core genome.

More details on these processes have now been added (page 6, lines 132-140).

Line 129: it would be informative to state the max and min % nucleotide distance among strains; were all strains clearly belonging to a single species?

Rather than using percent nucleotide distance, we describe differences measured in numbers of SNPs, as we believe this to be a more clear representation of the data (page 6, line 143). 89 strains showed sufficient coverage compared to the genome of K. pneumoniae MGH 78578, at an average of 91% (results from read mapping). The phylogeny based on concatenated seven MLST genes of all 89 strains in compared with K. variicola and K. pneumoniae MGH78578 showed that all 89 strains were highly related and belonged to K. pneumoniae species. This has been added (page 6, lines 132-140).

Line 139: but Bialek et al. EID 2014 recently showed ST14 and ST15 belong to distinct clonal groups.

This has been edited and the paper you mention has been cited "ST15 is an individual clonal group (CG) within the MLST clonal complex 14 (CC14) (Bialek-Davenet et al. 2014), an internationally dispersed clonal complex that has previously been associated with hospital-acquired infections (Hrabák et al, 2009)" (page 7, lines 161-163).

Line 140: is ST1559 phylogenetically closely related to known carbapenem-producing clones such as ST258 or ST11?

No. ST1559 is a single locus variant of ST152. This has been added for clarity (page 7, lines 167-168).

Lines 171 - 174: these molecular markers of lineage 1 are questionable as they are mobile elements or prone to HGT. Implications should be commented/could other strains harbouring these markers have been misidentified as lineage 1 by this PCR strategy?

We defined Outbreak Lineage 1 as ST15 possessing both the novel capsule and the pNDM-MAR plasmid. Though comparative genomics revealed additional specific genetic regions, these are all prone to HGT (i.e. prophage, plasmid). The use of capsule and resistance plasmids for K. pneumoniae typing and differentiation is of medical importance and has been applied previously (see Discussion). We are fully aware that these markers are prone to HGT. However, given our restricted time frame for PCR, we expect the impact of misidentification due to HGT to be minimal. This has been addressed in the limitations section of the Discussion (page 15, lines 376-382).

Line 172: Novel capsular type and Figure 3: surprisingly there is no wzy polymerase gene identified. Probably the hypothetical protein before wzx (why was this gene call wzxC)? Authors could hunt for conserved motives of wzy or use relaxed BLAST criteria, as this is a highly variable gene but always present in cps clusters.

This was an oversight on our part. The "O-antigen ligase" gene is indeed wzy, and this was confirmed by Pfam search to show the presence of the Wzy_C domain in the protein. This has been edited in Figure 3. WzxC has been edited to read wzx.

Line 198: 'were of capsule type 14': as deduced based on the genomic sequence? Based on which criteria? Or was antiserum used to confirm the serotype?

Yes, these were deduced based on the genome sequence of the whole capsule biosynthesis region (cps). This has been addressed (page 10, line 254). We did not have access to specific anti-serum.

Line 199: 'similar' is a bit vague; move this to next section.

This has been removed.

Line 204-205: yersiniabactin is not present in all Kp strains according to literature. What is meant with 'common': frequent?

Information regarding this aspect has been removed.

I found the yersinibactin phylogenetic analysis not really useful here and a bit out of scope; unless to say that both lineages acquired it independently, which is useful. Also it was unclear to me how the phylogeny was constructed: concatenated alignments of all CDSs of the cluster? If so, were they congruent among themselves or is there some kind of mosaicism?

This section has now been removed at the request of another reviewer.

Line 212: what is meant with 'contemporary'? Seems strange to me here.

This has been edited to read "uncharacterized" (page 10, line 237).

Line 213: 'orientation': organization meant? What about other cps clusters: are they not typically of low CG%?

This sentence has been removed

Line 217 'from Kp 342': supported by very high degree of similarity?

Yes, this is supported by a high degree of similarity (>99%). This has been added (page 10, line 242).

Line 220: not sure that kfu is a siderophore system (and also fec)

This has been edited to indicate that these are iron-acquiring systems (page 10, line 246).

Line 232: 'resistance plasmid': how many contigs/ fully covered? How do you know it is a single plasmid?

The number of contigs varies from strain to strain. Read mapping of Outbreak Lineage 1 strains showed that these covered the genome of pNDM-MAR. We hypothesize that the resistance plasmid in our study is closely related to pNDM-MAR. However, we were not able to resolve the complete assembly of the plasmid due to the limitations of short read sequencing.

Line 232: 'high degree': how much precisely?

Pairwise BLAST showed that the lineage 1's plasmid and pNDM-MAR are >99% in identity in homologous regions. We have now added this information to the text (page 11, line 267).

Line 235: 'excision' more likely possibly, but can it instead be gain of the NDM1 in the other strains?

We have edited "absent by an apparent perfect excision from pNDM-MAR in the remaining six isolates" (page 9, lines 210-211).

Line 244 'impacting polymixin susceptibility': possibly contradicts line 231/ what about colistin?

Mutations in PhoPQ or its regulators have been shown to confer resistance to polymyxin. We were not able to assess this experimentally but have suggested further studies that could focus on clarifying this. This has been changed to "the impact of these mutations on polymyxin susceptibility is not yet well understood" (page 11, line 276).

Line 260: it should be fair to quote Snitkin et al here.

We have now added additional discussion of the work by Snitkin et al. (page 12, line 297; page 15, line 391).

Lines 291-300: not totally clear to me...

This section has been edited to clarify our hypothesis surrounds the emergence and spread of Outbreak Lineage 1 (page 13, lines 326-339).

Line 312: 'these siderophores': which ones? Could quote here, Bachman work demonstrating how yersiniabactin is useful in complement to other siderophores.

This has been added (page 14, line 348).

Line 321 'cross sectional': what is meant (again!)?

This has been removed.

Line 322: how was CC14 determined if ST was not provided? CC definition is difficult in kp due to lack of clear discontinuities - so this is possibly a weak argument to say strains are closely related.

The cited study (Tada et al., 2013) did not provide specific ST, but instead use CC14. This also impeded us from drawing conclusions on their relatedness. Since they share multiple resistance determinants of similar origins, we hypothesize that the Outbreak Lineage 1 in this study and Tada's isolated strains are related or have shared the same resistant gene pool. This is only a hypothesis.

Line 347: Ramos et al was not an outbreak investigation; Snitkin et al is missing here.

We have added a reference to Snitkin et al. and clarified the relevance of that paper and that of Ramos et al. as they relate to our study (page 15, line 391).

Line 350 - 360: the authors turn a bit lyric about WGS here - I would tend to think that other typing methods could have been used e.g. a simple ERIC-PCR screen, MLST, PFGE, would all have identified/distinguished the 2 outbreak strains. I fully agree that WGS was key to dissect the outbreaks though. Maybe the authors would want to distinguish strain typing /surveillance on the one hand, and in-depth phylogenetic and genomic analyses rendered possible by WGS on the other hand.

This has been toned down to reflect these suggestions, and the final paragraph has been modified to discuss strain typing/ surveillance (page 16, lines 399-411).

Lines 419-420: no processing of the raw reads (quality...) was performed before assembly?

Reads were mapped to the reference genome Klebsiella pneumoniae MGH78578 by BWA, and QC stats were exported to graphs and manually assessed. Reads were mapped back to the assemblies. The average Phred quality score of the all sequencing read sets was 35 (33.7

– 37) (page 19, lines 472-476).

Lines 445-455: not sure I understood the process. What are the advantages of the strategy used here compared to classical protein cluster construction for accessory genome definition?

This strategy allows the accessory genome to be reassembled into contigs, which provides more information on the genetic organization of these elements. A protein clustering approach only produces a set of accessory genes without knowledge of how they are organized. We combined these two methods to best describe the accessory genome.

Line 466 'constant size demographic model': this seems inconsistent with the fact that outbreak strains were included, as a population expansion would have been expected. No?

We tested various demographic models in our initial analyses, including the exponential growth model and a Bayesian Skyline Plot (BSP) model. Although expectations suggested that we would see a rise in relative population size over the course of the outbreak, overlapping 95% HPDs for the BSP suggested that these differences were not significant. Additionally, Bayes Factor comparison of likelihoods did not estimate significant differences between the constant population size, exponential growth or BSP models. Therefore, we reverted to the simplest appropriate model (constant size) in an attempt to avoid over-parameterization. The lack of a signal for population growth is likely related in part to the short sampling timeframe and limited temporal signal in the data, and more sophisticated model priors likely would not be supported by the data and would add further uncertainty to this analysis.

Line 481: 'rule' without 'd' I guess. Were all genes of the HPI cluster considered together?

This section on the characterization of yersiniabactin operon diversity has been removed.

Lines 492-494: please provide polymerase/ reagent/ buffer/ kit used.

We have added this information into the methods (page 22, lines 555-556).

Figure 1 legend: 'MON' should be defined. Black arrow: only one lineage so no 's'. 'with a two mutations': ? Scale bar seems to imply many nucleotide differences among lineages. Is it really correct?

MON has been defined as month of isolation. The scale bar indicates the number of nucleotide changes per site, not the total number of differences.

Would be good to provide ST of all branches in Figure 1; possibly in Table S1? Are the branches closely related to ST15 and ST1559 (each have a small sister group) also ST15 and ST1559?

The STs of all isolates have been added to Table S1. While we agree that a visualization showing the ST for all branches might be interesting to include in Figure 1, we want to focus the reader primarily on the ST15 and ST1559 outbreak lineages here and think that it would be very difficult to maintain the clarity of the figure if we added additional information on all of the other STs.

The sister group of ST15 is ST14. The single strain most related to ST1559 is ST152 (page 7, line 167).

Figure 2 legend: OPD meaning? Why are there black dots right of the last black arrow?

We have now defined OPD (outpatient department) in the Figure Legend.

The black arrow indicates the last detected case of blood-borne NDM-1 positive K. pneumoniae. The figure legend has been edited as "Arrows signify the first and last occasions on which bla_{NDM-1} positive blood-borne Klebsiella pneumoniae Outbreak Lineage 1 strains were isolated."

Figure 3 legend: 'orientation': of the genes meant?

The word 'orientation' has now been deleted.

Figure 4 legend: only one bootstrap value shown it seems but plural used in legend?

This figure has been removed.

Figure 5: please name the genes in the legend. What is mph?

We have now added the functions of the genes to the Figure Legend.

Data Accessibility: were sequences submitted to public databases (eg novel capsule cluster)?

Yes all of the read information has been deposited into the European Nucleotide Archive (ENA) under the accession numbers ERR349747 to ERR349855. The assemblies of the two representatives of the two outbreaks have been registered as PRJEB8009 (lineage 1) and PRJEB8010 (lineage 2), and are awaiting final deposition. The capsule biosynthesis cluster has been submitted and has accession number LN714331.

2nd Editorial Decision 16 January 2015

Thank you for the submission of your revised manuscript to EMBO Molecular Medicine. We have now received the enclosed reports from the referees that were asked to re-assess it. As you will see the reviewers are now supportive, I am happy to say that your article will be accepted pending the minor text modifications suggested by the referee 1.

Please only resubmit your main article with the modifications in, and remove the line numbers.

I look forward to receiving a new revised version of your manuscript as soon as possible.

***** Reviewer's comments *****

Referee #2 (Remarks):

This new version of the manuscript "A high-resolution genomic analysis of multi-drug resistant hospital outbreaks of Klebsiella pneumoniae" by Chung The and colleagues is definitely improved and easier to read.
Most of my concerns have been properly addressed, and I believe the manuscript is now ready to be accepted.

I add few minor comments/typos that can be edited before final acceptance.

P7 : line 159 : resistant instead of non-susceptible

P10, line 240. K. pneumoniae strain 432 carries 3 different Type 6 secretion systems (locus I, II and III). Can the author specify to which of theses T6SS loci the one specifically found in lineage 1 corresponds?

P12, line 292 : "pneumoniae, is the use" instead of "and the use"

P20 line 507: add reference Lin et al 2012 for the reference strain 1084

P29 : description of panel c, use C in upper case.

Referee #4 (Remarks):

The authors have addressed my comments and other's in an appropriate way.
